# *quantro*: a data-driven approach to guide the choice of an appropriate normalization method

**DOI:** 10.1186/s13059-015-0679-0

**Published:** 2015-06-04

**Authors:** Stephanie C. Hicks, Rafael A. Irizarry

**Affiliations:** Department of Biostatistics and Computational Biology, Dana-Farber Cancer Institute, 450 Brookline Avenue, Boston, MA 02115-5450 USA; Department of Biostatistics, Harvard School of Public Health, 677 Huntington Avenue, Boston, MA 02115 USA

## Abstract

Normalization is an essential step in the analysis of high-throughput data. Multi-sample global normalization methods, such as quantile normalization, have been successfully used to remove technical variation. However, these methods rely on the assumption that observed global changes across samples are due to unwanted technical variability. Applying global normalization methods has the potential to remove biologically driven variation. Currently, it is up to the subject matter experts to determine if the stated assumptions are appropriate. Here, we propose a data-driven alternative. We demonstrate the utility of our method (*quantro*) through examples and simulations. A software implementation is available from http://www.bioconductor.org/packages/release/bioc/html/quantro.html.

**Electronic supplementary material:**

The online version of this article (doi:10.1186/s13059-015-0679-0) contains supplementary material, which is available to authorized users.

## Background

Multi-sample normalization techniques such as quantile normalization [1, 2] have become a standard and essential part of analysis pipelines for high-throughput data. These techniques transform the original raw data to remove unwanted *technical variation*. Technical variation can cause perceived differences between samples processed on high-throughput technologies, irrespective of the biological variation. These differences are typically due to changes in experimental conditions that are hard or impossible to control [3] and confusing them with biological variability can lead to false discoveries [4, 5].

Some of the first attempts at normalizing microarray data mimicked the use of so-called house-keeping genes [6] as was done by the established gene expression measurement technology that preceded microarrays. This approach did not work well in practice [7, 8]; therefore, data-driven approaches were developed, such as median correction [9, 10], variance-stabilizing transformation [11], locally weighted linear regression (loess) [12] and spline-based methods [13]. The general idea of these approaches is to assume that observed variability in global properties are due only to technical reasons and are unrelated to the biology of interest [2, 14]. Here we refer to these as *global adjustment* methods [15]. Examples of global properties include the total number of differentially expressed genes across groups, the median gene expression across genes and the statistical distribution of gene expression values. These types of assumptions are justified in many biomedical applications — for example, in gene expression studies in which only a minority of genes (or *targeted* set of genes) are expected to be differentially expressed. However, if, for example, a substantially higher percentage of genes are expected to be expressed in only one group of samples, it may not be appropriate to use global adjustment methods.

**Fig. 1:**
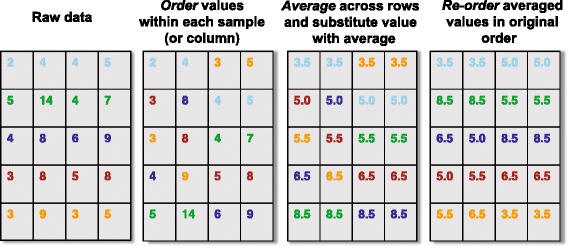
A schematic of quantile normalization. Quantile normalization is a non-linear transformation that replaces each feature value (row) with the mean of the features across all the samples with the same rank or quantile. To quantile normalize a raw high-throughput data set with multiple samples: (1) order the feature values within each sample; (2) for each feature, average across the rows; (3) substitute the raw feature value with the average; (4) re-order the transformed values by placing in the original order

Quantile normalization was originally developed for gene expression microarrays [1, 2] but today it is applied in a wide-range of data types, including genotyping arrays [16, 17], RNA-Sequencing (RNA-Seq) [18–20], DNA methylation [21], ChIP-Sequencing [22, 23] and brain imaging [24–26]. Quantile normalization is a global adjustment method that assumes the statistical distribution of each sample is the same. Normalization is achieved by forcing the observed distributions to be the same and the average distribution, obtained by taking the average of each quantile across samples, is used as the reference. This method has worked very well in practice but note that when the assumptions are not met, global changes in distribution that may be of biological interest will be wiped out and features that are not different across samples can be artificially induced [27]. A schematic of quantile normalization is provided in Fig. 1.

**Fig. 2:**
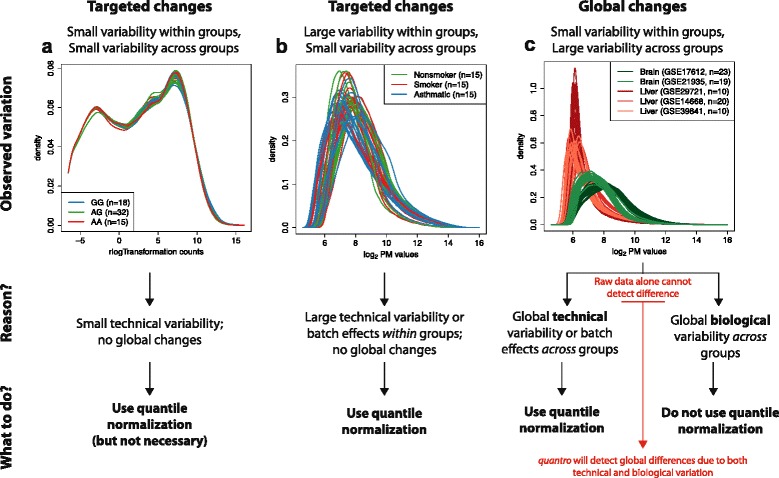
When to use quantile normalization? Examples of gene expression data with *targeted changes* and *global changes* in distributions across groups. **a** Transformed read counts from *n* = 65 RNA-Seq samples from the Yoruba (YRI) population and colored by genotype based on the eQTL rs7639979: GG (*blue*), GA (*green*) and AA (*red*). As no global differences in distributions were detected, this suggests quantile normalization is appropriate, but not necessary as there is a low level of variation within and between groups. **b** Raw perfect match (PM) values from *n* = 45 arrays comparing the gene expression of alveolar macrophages from nonsmokers (*green*), smokers (*red*) and patients with asthma (*blue*). No global differences in distributions were detected, which indicates quantile normalization is appropriate, as it will remove any platform-based technical variability or batch effects within groups. **c** Raw PM values from *n* = 82 arrays comparing brain and liver tissue samples. The samples are colored by tissue (brain [*red*] and liver [*green*]), and the shades represent different Gene Expression Omnibus IDs. The global differences in distributions detected across brain and liver tissues indicate quantile normalization is not appropriate. Global changes caused by technical variation (e.g., batch effects across groups) will also be detected by *quantro*, but raw data alone cannot detect this difference

Previously, the burden of deciding if these assumptions hold have been left to the experimentalist. Graphical assessments such as boxplots and density plots can be helpful, but they do not provide a quantitative measure of the variability. Here we propose a statistical test, referred to as *quantro*, for the assumptions of global adjustment methods, such as quantile normalization, that tests for global differences in distributions between groups of samples. Our test uses the raw unprocessed high-throughput data as input to calculate a test statistic comparing the variability of distributions within groups relative to between groups. If the variability between groups is sufficiently larger than the variability within groups, then this suggests there may be global differences in distributions between groups of samples and global adjustment methods may not be appropriate. We demonstrate the advantages of our method by applying it to several gene expression and DNA methylation datasets with *targeted* and *global* changes in distributions (Fig. 2). We define *global* changes as an abundance of differences between two or more sets of samples affecting the shape or the location shift of the distributions across groups caused by a biological or a technical source of variation and *targeted* changes as differences between sets of samples not affecting the shape or location shift of the distributions caused by a biological or a technical source of variation. To study the specific downstream improvements afforded by *quantro* we studied the specificity and sensitivity of differential expression estimates with a Monte Carlo simulation. Specifically, we studied how global normalization methods can lead to increased bias in downstream analyses, such as detecting differential methylation, when there are global differences in the distributions and how not applying appropriate normalization methods can lead to increased variance. We demonstrate how by guiding the choice of a normalization technique, our method provides an overall improvement in sensitivity and specificity.

## Results

### *quantro*: test for global differences in distributions between groups

Consider a set of raw high-throughput data *X*_*ik*_ representing *i* ∈ (1, …, *n*_*k*_) samples in each of the *k* ∈ (1, …, *K*) groups (*n*_*T*_ total samples) from a gene expression or DNA methylation experiment. We assume *X*_*ik*_ has some common distribution (*X*_*ik*_ ∼ ℱ_*k*_) where ℱ_*k*_ is the theoretical distribution for the *k*^*th*^ group. We define ${F}_{ik}^{-1}$ as the observed quantile distribution for the *i*^*th*^ sample in the *k*^*th*^ group. As a first step, we use an ANOVA to test if the average of the medians of the distributions are different across groups and median normalize the samples accordingly. Let ${\overline{F}}_{.k}^{-1}=\frac{1}{n_k}{\displaystyle {\sum}_{i=1}^{n_k}{F}_{ik}^{-1}}$ be the quantile distribution averaged across all samples in the *k*^*th*^ group and let ${\overline{F}}_{..}^{-1}=\frac{1}{K}\frac{1}{n_k}{\displaystyle {\sum}_{k=1}^K{\displaystyle {\sum}_{i=1}^{n_k}{F}_{ik}^{-1}}}$ be the quantile distribution averaged across all samples and groups.

To quantify the differences between two distributions, we use Mallow’s distance [28], which is defined as the distance between two probability distributions over a region (Eq. S1 in Additional file 1). We define the *total variance* of the distributions as the sum of squared differences between *F*_*ik*_^− 1^ and ${\overline{F}}_{..}^{-1}$ using Mallow’s distance (in the case where *p* = 2) as:

$$ S{S}_{total}={\displaystyle {\sum}_{k=1}^K{\displaystyle {\sum}_{i=1}^{n_k}{{\displaystyle \int \left({F}_{ik}^{-1}-{\overline{F}}_{..}^{-1}\right)}}^2}} $$

The *total variance* can be decomposed (Eqs. S2–7 in Additional file 1) into the variance between groups (*SS*_*between*_) and the variance within groups (*SS*_*within*_):

$$ {\displaystyle \sum_{k=1}^K}{\displaystyle \sum_{i=1}^{n_k}}{\displaystyle \int }{\left({F}_{ik}^{-1}-{\overline{F}}_{..}^{-1}\right)}^2 = {\displaystyle \sum_{k=1}^K}{\displaystyle \sum_{i=1}^{n_k}}{\displaystyle \int }{\left({\overline{F}}_{.k}^{-1}-{\overline{F}}_{..}^{-1}\right)}^2 + {\displaystyle \sum_{k=1}^K}{\displaystyle \sum_{i=1}^{n_k}}{\displaystyle \int }{\left({F}_{ik}^{-1}-{\overline{F}}_{.k}^{-1}\right)}^2 $$

We propose using a data-driven test statistic, referred to as *F*_*quantro*_, to test for global differences in the distributions between the *K* groups. The null hypothesis is that there are no global differences in the distributions between the groups and the alternative hypothesis is that at least one group is different from the rest.

$$ \begin{array}{l}{H}_0:\ {\mathrm{\mathcal{F}}}_1={\mathrm{\mathcal{F}}}_2=\cdots ={\mathrm{\mathcal{F}}}_K\\ {}{H}_a:\ {\mathrm{\mathcal{F}}}_i\ne {\mathrm{\mathcal{F}}}_j\ \mathrm{f}\mathrm{o}\mathrm{r}\ \mathrm{at}\ \mathrm{least}\ \mathrm{o}\mathrm{ne}\ i,\ j\end{array} $$

If there are no global differences in the distributions between the groups (due to technical or biological variation), we can apply a global adjustment method, such as quantile normalization, to remove any unwanted technical variation. If there are global differences in the distributions between the groups, quantile normalization may not be an appropriate normalization technique depending on the source of variation (technical or biological variation).

The *F*_*quantro*_ test statistic (Eq. S8 in Additional file 1) is a ratio of the mean squared error between groups (*MS*_*between*_) to the mean squared error within groups (*MS*_*within*_):

$$ {F}_{quantro}=\frac{M{S}_{between}}{M{S}_{within}}=\frac{S{S}_{between}/\left(K-1\right)}{S{S}_{within}/\left({n}_T-K\right)} $$

We use permutation testing to assess the statistical significance of *F*_*quantro*_ and reject the null hypothesis if the *p* value (Eq. S9 in Additional file 1) from the permutation test is less than some α significance level.

### Targeted and global changes in gene expression

We applied *quantro* to several publicly available gene expression datasets based on both microarray and RNA-Seq platforms (Table S1 in Additional file 1) to investigate *targeted* and *global* differences in distributions across groups. We used an α = 0.05 significance level as the threshold to test for *global* changes in the distributions across groups. Examples of targeted changes in distributions across groups are the gene expression of samples from the Yoruba (YRI) population stratified by genotype based on an expression quantitative trait loci (eQTL) (*p* = 0.917; Fig. 2a; Figure S1 in Additional file 1), samples from two inbred mouse strains (*p* = 0.245; Figure S2 in Additional file 1), samples of alveolar macrophages from nonsmokers, smokers and patients with asthma (*p* = 0.562; Fig. 2b; Figure S3 in Additional file 1), samples of bronchial brushings from individuals with and without chronic obstructive pulmonary disease (*p* = 0.218; Figure S4 in Additional file 1) and samples from two regions of the brain in patients with Parkinson’s disease (*p* = 0.264; Figure S5 in Additional file 1). In all of the above examples, quantile normalization is considered appropriate because no global differences in the distributions across groups were detected at the α = 0.05 significance level.

When comparing the gene expression of two tissues, we found striking global differences in the distributions between brain and liver tissues (*p* = 0.004; Fig. 2c; Figure S6 in Additional file 1). We considered multiple studies from the Gene Expression Omnibus (GEO) to represent each tissue to prevent batch effects [29] of different studies from GEO being confounded with differences in tissues. We also compared the gene expression of normal and tumor samples. We obtained multiple studies from GEO and found global differences in the distributions between the normal and tumor samples of lung (*p* < 0.001; Fig. 2d), breast (*p* < 0.001), prostate (*p* < 0.001), thyroid (*p* < 0.001), stomach (*p* < 0.001) and liver tissues (*p* = 0.044) (Figures S7–12 in Additional file 1). We also found global changes in the distributions of liver tissues between four groups of patients (control, healthy obese, steatosis and nash samples) from a study investigating the gene expression of non-alcoholic fatty liver disease (*p* = 0.004; Figure S13 in Additional file 1).

### Targeted and global changes in DNA methylation

**Fig. 3:**
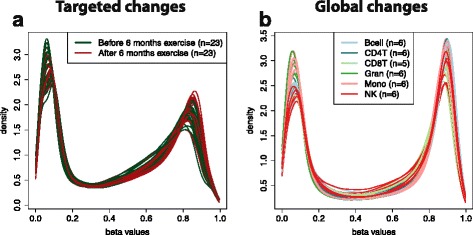
Biological variation in distributions of raw DNA methylation microarrays. **a** Example of *targeted changes* in distributions: raw beta values from *n* = 46 arrays comparing adipose tissue samples from healthy men before and after 6 months of exercise. **b** Example of *global changes* in distributions: raw beta values from *n* = 35 arrays comparing six purified cell types from whole blood: CD14+ monocytes (*Mono*), CD19+ B cells (*Bcell*), CD4+ T cells (*CD4T*), CD56+ natural killer cells (*NK*), CD8+ T cells (*CD8T*), and granulocytes (*Gran*)

In addition to gene expression, we considered three publicly available DNA methylation data sets. We detected no global differences in distributions of adipose tissues from patients before and after six months of exercise (*p* = 0.132; Fig. 3a; Figure S14 in Additional file 1) and pancreatic tissues from non-diabetic and type 2 diabetes (*p* = 0.069; Figure S15 in Additional file 1). In contrast, *quantro* detected global differences in the distributions across six purified cell types from whole blood (*p* < 0.001; Fig. 3b; Figure S16 in Additional file 1), which may be relevant for the studies estimating the cell composition of whole blood using DNA methylation [30, 31].

### *quantro* improves the accuracy of detecting differentially methylated CpGs

Here we evaluate the performance of global normalization methods in the context of *targeted* and *global* changes in distributions with the goal of detecting differentially methylated CpGs. We performed a Monte Carlo simulation study to illustrate how the use of global normalization methods, such as quantile normalization, is not always appropriate and the *F*_*quantro*_ test statistic can guide the choice of normalization. For the simulation study, we simulate DNA methylation arrays with a goal of detecting differentially methylated CpGs, but note these results also translate for differential gene expression. We compare naively using quantile normalization to using *quantro* to guide the decision of using either quantile normalization or no normalization to assess the cost of using global normalization methods in the context of distributions with global differences.

If there is only a minority of differentially methylated CpGs, quantile normalization reduces the bias and mean squared error (MSE) in detecting true differences between groups of samples because it removes unwanted technical variation (Figures S21 and S22 in Additional file 1). As the number of differentially methylated CpGs increases, quantile normalization will remove both the unwanted technical and interesting biological variation, resulting in higher bias and MSE when detecting differential methylation. In contrast, the use of *quantro* detects these global differences and therefore reduces the bias and MSE compared with using quantile normalization (Figures S21 and S22 in Additional file 1). Similarly, the number of false discoveries is reduced when using *quantro* to guide the normalization choice in the case when there are global differences between groups. For example, when considering a 450K DNA methylation array if there are only a small number of differentially methylated CpGs (1 % of CpGs or 4500 CpGs), *quantro* and quantile normalization are comparable in the number of false discoveries (873 and 873, respectively), but if there are global differences in the distributions between groups (10 % of CpGs or 45,000 CpGs), *quantro* is able to detect those global differences and reduce the number of false discoveries compared with quantile normalization (4887 and 6583, respectively) (Figure S23 in Additional file 1). Using *quantro* gives researchers a data-driven tool to test if global normalization methods are appropriate, such as quantile normalization, which can result in larger bias, MSE and more false discoveries when detecting differentially methylated CpGs in the context of global differences in distributions.

In addition, we considered the true positive rate and false positive rate of using quantile normalization and using *quantro* to guide the choice of normalization while varying the threshold of the number of top differentially methylated CpGs selected. If there are only a small number of differentially methylated CpGs, quantile normalization and *quantro* are comparable in performance, but when the proportion of differentially methylated CpGs increases, quantile normalization fails to detect global differences between the groups, resulting in lower sensitivity and specificity (Figure S24 in Additional file 1). Using *quantro* as a tool to determine which type of normalization approach to employ results in higher sensitivity and specificity when detecting true differentially methylated CpGs compared with naively using quantile normalization.

## Discussion

The advent of high-throughput technologies brought the opportunity for researchers to investigate and assess biological variability at the genomic level, but it also introduced unwanted technical variability that can cause perceived differences between samples processed on high-throughput technologies, irrespective of the biological variation. These differences may be due to differences in the way the samples were processed (such as batch effects) or to platform-dependent technical variation. Because global changes in distributions between groups can be caused by both technical variation and biological variation, it is important to note that our test statistic *F*_*quantro*_ will detect global differences caused by both technical variation (e.g., batch effects) and biological variation. Data alone cannot determine if global changes are caused by technical variation or biological variation (Fig. 2), but *quantro* offers researchers a new tool to detect when there are global changes in distributions across groups.

As *quantro* assess the assumptions of global normalization methods, it is important to note other global normalization methods such as VSN [11], Loess [12] and the trimmed mean method [32] make different assumptions about the true biological variability [33]. For example, the trimmed mean method assumes most genes are not differentially expressed and uses a global linear scaling factor for normalization. Similarly, Loess uses local scaling factors in a moving window. In contrast to scaling factors, VSN is based on a slightly different assumption that the variance is constant and transforms the raw data such that the variance is constant across expression levels, reducing the variability observed in regions with low expression. The method introduced here, *quantro*, is not a normalization method, but rather it tests the assumptions of global normalization methods that assume there are no global differences in the distributions to guide the choice or whether or not global normalization methods are appropriate.

Here, we have shown if there are global changes in the distributions across a set of groups, normalization methods with *global adjustments* may not be appropriate depending on the type and source of variation. If global adjustment methods are not appropriate, other methods, such as *application-specific* methods [15], can be used. These are normalization methods where the adjustments are directly incorporated into the experiment or main analysis. Examples of these methods include the use of positive and negative control genes, the use of spike-in controls and explicitly modeling known or unknown effects of unwanted variation in a linear model (see Section 5 in Additional file 1 for more a more detailed discussion on application-specific methods). It is important to note that some of the application-specific methods, such as SVA [34] and PEER [35], are examples of a method that will likely remove true global differences as these are often captured by the first principal components. In addition, methods that rely on control genes, such as RUV [15], are similar to other forms of global normalization methods such as subset quantile normalization.

Previous studies have evaluated and discussed normalization methods with and without global adjustments [2, 15, 32, 36], but the decision of which type of normalization method to use depends on the outcome of interest. For example, a recent study [27] discussed the use of normalization procedures in global gene expression analysis comparing two schematics: targeted changes in gene expression and global changes in gene expression such as transcriptional amplification [37] or transcriptional shutdown [38]. Not surprisingly, the authors show normalization methods with global adjustments are not appropriate if the total RNA is not the same across the samples. In this case, if normalization is performed at the experimental level (introducing similar amounts of RNA into the assay from the two groups with global changes), then we suggest using control genes or spike-in controls as no differences between the distributions will be detected (Figure S25 in Additional file 1). However, for the great majority of studies such strategies are not available. Furthermore, if one knows a priori that most genes are differentially expressed, then high-throughput technologies may not be the optimal tool as these technologies are mainly used and have been optimized for finding specific genes that are differentially expressed between groups of samples.

## Conclusions

Normalization methods with *global adjustments* are widely used for data analysis in genomics, but rely on assumptions about the data generation process that are not appropriate in certain contexts. To the best of our knowledge, there is no quantitative method available to assess if the stated assumptions are appropriate or not, leaving the decision up to subject matter experts. Our method is the first to provide a data-driven solution to test the assumptions of global normalization methods. We have demonstrated the utility of our method by applying it to several gene expression and DNA methylation datasets, revealing examples of both targeted and global changes in distributions across groups, such as the global changes in distributions detected between the gene expression of brain and liver tissues. We demonstrated that global normalization methods can lead to increased bias and MSE in downstream analyses when there are global differences in distributions and *quantro* can detect when global normalization methods are not appropriate, which can prevent removing potentially interesting biological variation. We have implemented our method into the *quantro* R-package providing researchers a tool to test the assumptions of global normalization methods in the analysis of their own data.

## Materials and methods

### Data analysis

The method introduced here has been implemented into the *quantro* R-package available on Bioconductor. We used permutation testing to assess the statistical significance of the test statistic and distributed the computations across multiple cores to increase the speed. To test for global differences in distributions between groups of samples from high-throughput data sets, we applied *quantro* to several publicly available gene expression and DNA methylation data sets. Table S1 in Additional file 1 contains a list of all the data sets. For this analyses, we use the α = 0.05 significance level as the threshold to detect *global* changes in the distributions across groups.

To compare the gene expression on microarrays of cancer samples and brain and liver tissues, we considered multiple studies from GEO [39] to represent each tissue to prevent batch effects of different studies from GEO being confounded with differences between cancer samples or between tissues. For the gene expression samples using microarrays, we extracted the raw perfect match (PM) values from the CEL files using the *affy* R/Bioconductor package [40]. To visualize the true biological variation in the experimentally normalized samples from Lovén et al. [27], we divided the raw PM values by the sample mean of the PM values across the spike-ins on the log_2_ scale. For the gene expression samples using RNA-Seq, we used the rlogTransformation provided in the *DESeq2* R/Bioconductor package [41] to transform the raw counts to the log_2_ scale, which reduces the variability in the low counts, but other transformations can be used such as the Variance Stabilizing Transformation (VST) in the *DESeq2* package. The RNA-Seq data were obtained from ReCount [42], which pre-processes the raw sequencing data and provides a table of raw counts for each gene. We removed all the rows with zero counts across all the samples. For the DNA methylation samples using microarrays, we used the *minfi* R/Bioconductor package [43]. We extracted the raw methylated and unmethylated signal using and computed the ‘beta’-values using Illumina’s default setting of the *offset* parameter equal to 100.

### Details for simulation studies

We developed an R package, referred to as *quantroSim* (Section 3 in Additional file 1), which is available on GitHub, to simulate gene expression and DNA methylation data, but here we just focus on DNA methylation. To simulate samples on a microarray platform technology, we use the Langmuir adsorption model [44] to model the chemical saturation in the hybridization of the probes. Each of the simulation studies considered two groups with five samples each (total of ten samples).

With the goal of detecting differentially methylated CpGs, we compared the performance of *quantro* to the naïve approach of always using quantile normalization where *quantro* uses the *F*_*quantro*_ test statistic to decide if quantile normalization is appropriate (no normalization otherwise; Section 4 in Additional file 1). For the permutation testing in *quantro*, we used 100 permutations and a cutoff threshold of α = 0.05, unless specified otherwise. After normalization, the difference between the group means were estimated and the top differentially methylated probes were found using a *t*-test.

We assessed the relative bias (bias from *quantro* to the bias from quantile normalization) and relative MSE while varying the cutoff threshold from *quantro* and for a fixed threshold at α = 0.05. We simulated DNA methylation samples with a varying proportion of differentially methylated CpGs between the two groups and a varying level of technical variation (see Sections 3 and 4 in Additional file 1 for more details).

To select a list of top differentially methylated probes, we adjusted the *p* values from a *t*-test using the Benjamini and Hochberg adjustment to correct for multiple testing. The number of false discoveries was calculated using as the number of incorrectly selected probes from a given set of top differentially methylated probes. The true positive rate was calculated as the number of correctly selected probes from the set of true differentially methylated probes. In contrast, the false positive rate was calculated as the number of incorrectly selected probes from the set of probes that are not differentially expressed.

### Software

The R-package *quantro* implementing our method is available in Bioconductor 3.1 [45]) (software license GNU GPL 3.0) and the *quantroSim* R-package to simulate gene expression and DNA methylation data is available on GitHub [46].

## Additional file

Additional file 1: **Supplementary materials are available in a single pdf.** All scripts containing the code for these analyses are available on GitHub [47].

## Abbreviations

GEO: Gene Expression Omnibus
MSE: mean squared error
PM: perfect match
