# Supplementary material for: quantro: a data-driven approach to guide the choice of an appropriate normalization method
Source: Genome Biol. 2015 Jun 4;16(1):117. doi: 10.1186/s13059-015-0679-0 (PMC4495646; doi:10.1186/s13059-015-0679-0)
Supplement: Supplementary file 1 — Supplementary materials are available in a single pdf. All scripts containing the code for these analyses are available on GitHub [47]. [file 13059_2015_679_MOESM1_ESM.pdf]

Supplementary Material to: When to use Quantile Normalization?

Stephanie C. Hicks[1,2] and Rafael A. Irizarry[1,2]

[1]Department of Biostatistics and Computational Biology, Dana-Farber Cancer Institute
[2]Department of Biostatistics, Harvard School of Public Health

February 18, 2015

Contents

1 `quantro`: An R-package to test for global differences in distributions across groups 2
1.1 Deriving the test statistic $F_{quantro}$ . . . 2
1.2 Assessing the statistical significance of $F_{quantro}$ . . . 4

2 Description of high-throughput data used 5
2.1 Gene expression . . . 6
2.1.1 RNA-Sequencing . . . 6
2.1.2 Microarrays . . . 8
2.2 DNA methylation . . . 21
2.2.1 Microarrays . . . 21

3 `quantroSim`: An R-package to simulate gene expression and DNA methylation data 25
3.1 Simulating gene expression samples . . . 25
3.1.1 Microarrays . . . 26
3.2 Simulating DNA methylation samples . . . 28
3.2.1 Microarrays . . . 30

4 Simulation Study: Assessing the Performance of `quantro` 34
4.1 Performance metrics . . . 34
4.2 Bias-Variance trade-off . . . 35
4.2.1 Bias-Variance trade-off as a function of the cutoff used by `quantro` . . . 36
4.2.2 Bias-Variance trade-off using `quantro` threshold of $\alpha = 0.05$ . . . 37
4.3 Number of false discoveries . . . 38
4.4 Receiver operating characteristic curves . . . 39

5 Alternatives to Global Normalization Methods 41
5.1 Application-specific normalization methods . . . 41
5.1.1 Impact of experimental normalization in the context of global changes in gene expression . . 41

6 Software Available 43

1 `quantro`: An R-package to test for global differences in distributions across groups

The `quantro` R-package can be used to test for global differences in distributions between groups to guide the choice of whether it is appropriate to use global normalization methods, such as quantile normalization. Our method uses the raw unprocessed high-throughput data and computes a test statistic to compare the variability of distributions within each group to the variability of distributions between the groups. If the variability between the groups is sufficiently larger than the variability within each group, then there may be global differences between the groups of distributions suggesting quantile normalization may not appropriate, depending on the type and source of variation. The main function `quantro()` will perform two tests:

1. An ANOVA to test if the medians of the distributions are different across groups. Differences across groups could be attributed to unwanted technical variation (such as batch effects) or real global biological variation. This is a helpful step for the user to verify if there is any technical variation unaccounted for.

2. A test for global differences between the distributions across groups which returns a test statistic called `quantroStat`. This test statistic is a ratio of two variances (similar to the idea of ANOVA): the variability of the distributions within groups relative to the variability between groups. If the variability between groups is sufficiently larger than the variability within groups, then this suggests global adjustment methods may not be appropriate. As a default, we perform this test on the median normalized data, but the user may change this option.

1.1 Deriving the test statistic $F_{quantro}$

Assume we have a set of n_T samples representing K groups (e.g. $K = 2$ if case/control comparison). Within the kth group, assume there are n_k samples. We let each sample have N observations. Assume the distribution representing the ith sample in the kth group ($\mathcal{F}_{ik}$) has some common distribution ($\mathcal{F}_k$). Using the raw observed data, we apply a median normalization to the set of samples by removing each sample median. Let the median normalized data be given by $\mathbf{X}$ where $(X_{ik})_j$ represents the jth observation (row) from the ith individual in the kth group (column).

We assume

$$(X_{ik})_j \sim \mathcal{F}_{ik} \text{ where } E[(X_{ik})_j] = \mu_k \text{ and } Var[(X_{ik})_j] = \sigma^2$$

and define $F_{ik}^{-1}(u)$ as the quantile function (or inverse distribution function) of $\mathbf{X}_{ik}$ where $u \in [0, 1]$. Consider

$$\bar{F}_{\cdot k}^{-1}(u) = \frac{1}{n_k} \sum_{i=1}^{n_k} F_{ik}^{-1}(u) \quad \text{and} \quad \bar{F}_{\cdot\cdot}^{-1}(u) = \frac{1}{K} \frac{1}{n_k} \sum_{k=1}^{K} \sum_{i=1}^{n_k} F_{ik}^{-1}(u)$$

where $\bar{F}_{\cdot k}^{-1}(u)$ represents the uth quantile averaged across samples in the kth group and $\bar{F}_{\cdot\cdot}^{-1}(u)$ represents the uth quantile averaged across all samples and groups.

Under the null hypothesis of no global differences in distributions between groups, we formally define our null and alternative hypotheses in terms for the quantile functions for each of K groups:

$$H_0: \ \mathcal{F}_1 = \mathcal{F}_2 = \ldots = \mathcal{F}_K$$

$$H_a: \ \mathcal{F}_i \neq \mathcal{F}_j \text{ for at least one } i, \ j$$

To quantify the variability within and between groups of distributions, we define the "total variance" as the sum of squared differences (SS_{total}) between F_{ik}^{-1} and $\bar{F}_{\cdot\cdot}^{-1}$ using Mallow's distance. Mallow's distance [1] (also more generally known as Earth mover's distance or the Wasserstein metric) is defined as the distance between two probability distributions (say F, $G \in \mathbb{R}$) over a region

$$M_p(F, G) = \Big(\int_0^1 |F^{-1}(u) - G^{-1}(u)|^p du \Big)^{(1/p)} \tag{1}$$

where F^{-1} and G^{-1} represent the quantile functions corresponding to the distributions F and G. Here, we show the "total variance" (or sum of squares) of all the distributions (SS_{total}) can be written as the sum of the "variance between groups" ($SS_{between}$) and the "variance within groups" (SS_{within}):

$$\begin{aligned}
\text{SS}_{total} &= \sum_{k=1}^{K}\sum_{i=1}^{n_k} \Big(M_2(F_{ik}^{-1}, \bar{F}_{\cdot\cdot}^{-1}) \Big)^2 && (2)\\
&= \sum_{k=1}^{K}\sum_{i=1}^{n_k} \int_0^1 (F_{ik}^{-1}(u) - \bar{F}_{\cdot\cdot}^{-1}(u))^2 du && (3)\\
&= \sum_{k=1}^{K}\sum_{i=1}^{n_k} \int_0^1 \Big[(\bar{F}_{\cdot k}^{-1}(u) - \bar{F}_{\cdot\cdot}^{-1}(u)) + (F_{ik}^{-1}(u) - \bar{F}_{\cdot k}^{-1}(u)) \Big]^2 du && (4)\\
&= \sum_{k=1}^{K}\sum_{i=1}^{n_k} \int_0^1 (\bar{F}_{\cdot k}^{-1}(u) - \bar{F}_{\cdot\cdot}^{-1}(u))^2 du + \sum_{k=1}^{K}\sum_{i=1}^{n_k} \int_0^1 (F_{ik}^{-1}(u) - \bar{F}_{\cdot k}^{-1}(u))^2 du && (5)\\
&= \sum_{k=1}^{K}\sum_{i=1}^{n_k} \Big(M_2(F_{\cdot k}^{-1}, \bar{F}_{\cdot\cdot}^{-1}) \Big)^2 + \sum_{k=1}^{K}\sum_{i=1}^{n_k} \Big(M_2(F_{ik}^{-1}, \bar{F}_{\cdot k}^{-1}) \Big)^2 && (6)\\
&= \text{SS}_{between} + \text{SS}_{within} && (7)
\end{aligned}$$

where the cross product terms cancel out because $\sum_{i=1}^{n_k} (\bar{F}_{\cdot k}^{-1}(u) - \bar{F}_{\cdot\cdot}^{-1}(u)) = 0$.

We propose using a data-driven test statistic, referred to as $F_{quantro}$, to test for global differences in distributions across groups. This test statistic is a ratio of the variability in the distributions between groups ($MS_{between}$) to the

to the variability in the distributions within groups (MS_{within}):

$$F_{quantro} = \frac{MS_{between}}{MS_{within}} = \frac{SS_{between}/(K-1)}{SS_{within}/(n_T - K)} \tag{8}$$

1.2 Assessing the statistical significance of $F_{quantro}$

To assess statistical significance, we use permutation testing. We permute the variable defining the group level information B times and re-calculate the test statistic for each permuted sampled ($F^b_{quantro}$). The p-value from the permutation test is calculated as

$$p = \sum_{b=1}^{B} I_{[F^b_{quantro} > F_{quantro}]} \tag{9}$$

Using some α significance level, if $p \geq \alpha$, then we fail to reject H_0. If $p < \alpha$, then we would reject H_0.

2 Description of high-throughput data used

To investigate global differences in distributions between groups of samples from high-throughput data sets, we considered several publicly available gene expression and DNA methylation data sets. Table 1 contains a list of the data sets used for this analysis. We considered several experimental design scenarios such as comparing samples across tissues, populations, cell types, and outcomes such as 'normal' and 'tumor' samples. We used a significance level of $\alpha = 0.05$, but refer the reader to the simulation studies in Section 4 for a more detailed discussion on other choices for α.

Table 1: Summary of high-throughput data sets used

	Comparison	Name of data set	# Samples	Reference/GEO accession ID
gene expression	eQTL status in YRI: rs7639979	pickrellRNASeq	69	Ref [2]. Available on Recount [3][1]
	Two mouse strains: B6, D2	mouseStrainsRNASeq	21	Ref [4]. Available on Recount [3][1]
	Nonsmokers, smokers, asthma	alveolarSmokingAffyData	45	Ref [5]. GEO: GSE2125
	Disease status: COPD	lungCOPDAffyData	238	Ref [6]. GEO: GSE37147
	Two regions of the brain	brainParkinsonsAffyData	22	Ref [7]. GEO: GSE19587
	Two tissues: brain, liver	brainLiverAffyData	82	GSE17612, GSE21935, GSE14668, GSE29721, GSE38941
	Disease status: normal, tumor	lungCancerAffyData	444	GSE18842, GSE19188, GSE19804, GSE10445, GSE12667, GSE2109
	Disease status: normal, tumor	breastCancerAffyData	931	GSE10780, GSE10810, GSE29431, GSE30010, GSE2109, GSE5460, GSE653, GSE9195
	Disease status: normal, tumor	prostateCancerAffyData	167	GSE17951, GSE32448, GSE2109
	Disease status: normal, tumor	thyroidCancerAffyData	98	GSE29265, GSE33630, GSE2109
	Disease status: normal, tumor	stomachCancerAffyData	82	GSE13911, GSE2109
	Disease status: normal, tumor	liverCancerAffyData	176	GSE14668, GSE29721, GSE38941, GSE2109, GSE9829;GSE9843
	Disease status: NAFLD	liverNAFLDAffyData	73	Ref [8]. GEO: GSE48452
	Two treatments: low and high levels of c-Myc	mycAffyData	4	Ref [9]. GEO: GSE40784
DNA methylation	Disease status: before, after 6 months exercise	adiposeExerciseMethyl	46	Ref [10]. Available online[2]
	Disease status: non-diabetic, Type 2 diabetes	pancreaticT2DMethyl	49	Ref [11]. Available online[2]
	Six cell types from whole blood	cellcompMethyl	36	Ref [12]. GEO: GSE35069

[1] http://bowtie-bio.sourceforge.net/recount/index.shtml
[2] http://www.ludc.med.lu.se/research-units/epigenetics-and-diabetes/published-data

2.1 Gene expression

2.1.1 RNA-Sequencing

We considered several examples of gene expression using RNA-Sequencing (RNA-Seq). The following RNA-Sequencing data sets are available from ReCount [3] at `http://bowtie-bio.sourceforge.net/recount/index.shtml`. ReCount pre-processes the raw sequencing data and provides a table of raw counts for each gene. We removed all the rows with zero counts reported across all the samples. We used the `rlogTransformation()` function provided in the `DESeq2` [13] R/Bioconductor package to transform the raw counts to the $\log_2$ scale. Note: the `VST()` function in the `DESeq2` package could also be used.

pickrellRNASeq. We considered a study originally performed to identify expression quantitative trait loci (eQTLs) using RNA-Seq data [2]. For this analysis, we considered one of the eQTLs identified in the YRI population (rs7639979) which is discussed in [2]. This eQTL stratifies the $n = 69$ samples in the YRI population by the genotypes GG ($n = 18$), GA ($n = 32$), AA ($n = 15$) and NN ($n = 4$). We removed the four samples with the missing NN genotype for this analysis. The densities and box plots of the `rlogTransformation` counts are shown in Figure 1 and colored by genotype: GG (blue), GA (green) and AA (red). We tested for global differences in the distributions across the groups stratified by genotype using `quantro`. We assessed the statistical significance of the test statistic ($F_{quantro} = 0.376$) using permutation testing and report there were no global differences detected at the $\alpha = 0.05$ level between the distributions of the groups ($p = 0.917$).

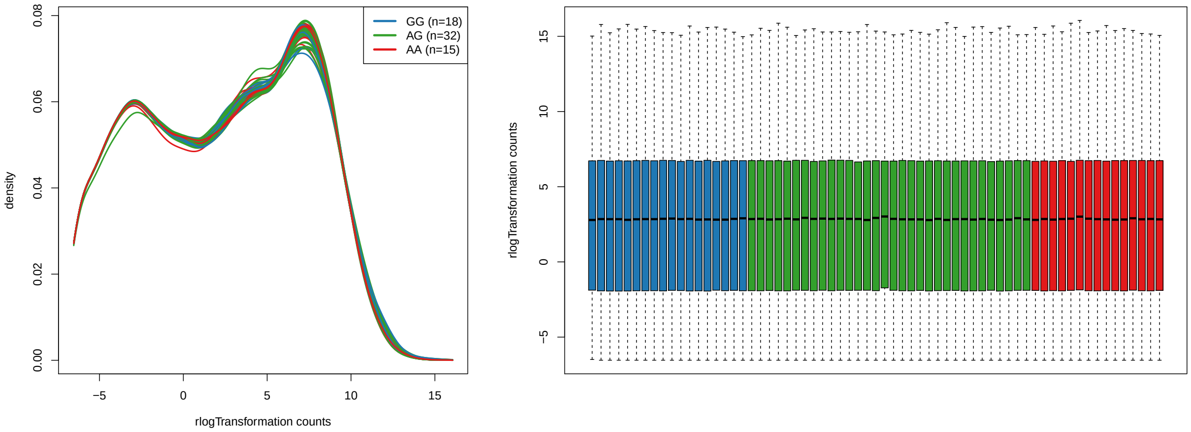

Figure 1: Densities and box plots of the `rlogTransformed` counts from RNA-sequencing samples in *pickrellRNASeq*. The plots use $n = 65$ samples from the groups based on the eQTL identified in the YRI population (rs7639979) and colored by by genotype: GG (blue), GA (green) and AA (red). Using `quantro`, we report no global differences detected at the $\alpha = 0.05$ level between the distributions using the eQTL status ($p = 0.917$).

mouseStrainsRNASeq. We considered a study originally performed to compare the gene expression of two inbred mouse strains using RNA-Seq data [4]. For this analysis, we considered $n = 21$ samples from the two inbred mouse strains: B6 strain ($n = 10$) and D2 strain ($n = 11$). The densities and box plots of the `rlogTransformation` counts are shown in Figure 2 and colored by mouse strain: B6 (green) and D2 (red). We tested for global differences in the distributions between the mouse strains using `quantro`. We assessed the statistical significance of the test statistic ($F_{quantro} = 1.215$) using permutation testing and report there were no global differences detected at the $\alpha = 0.05$ level between the distributions of mouse strains ($p = 0.245$).

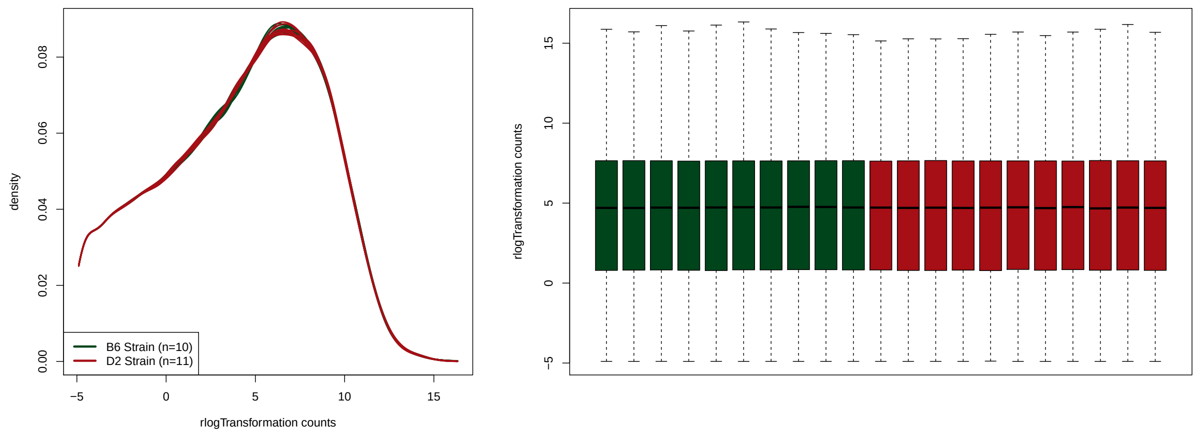

Figure 2: Densities and box plots of the `rlogTransformed` counts from RNA-sequencing samples in *mouseStrainsRNASeq*. The plots use $n = 21$ samples colored by by mouse strain: B6 strain (green) and D2 strain (red). Using `quantro`, we report no global differences detected at the $\alpha = 0.05$ level between the two mouse strains ($p = 0.245$).

2.1.2 Microarrays

We used the *affy* R/Bioconductor package [14] to analyze Affymetrix GeneChip arrays. We extract the raw Perfect Match (PM) probes from the original CEL files.

alveolarSmokingAffyData. We examined 45 Affymetrix Gene Chip arrays (GEO accession GSE2125) [5] which compared the gene expression of alveolar macrophages of $n = 45$ from 15 smokers, 15 nonsmokers, and 15 subjects with asthma (disease control). The densities and box plots of the raw PM values are shown in Figure 3 and colored by disease status: Nonsmoking (green), Smoking (red), Asthma (blue). We tested for global differences in the distributions between the three groups using `quantro`. We assessed the statistical significance of the test statistic ($F_{quantro} = 0.601$) using permutation testing and report there were no global differences detected at the $\alpha = 0.05$ level between the groups ($p = 0.562$).

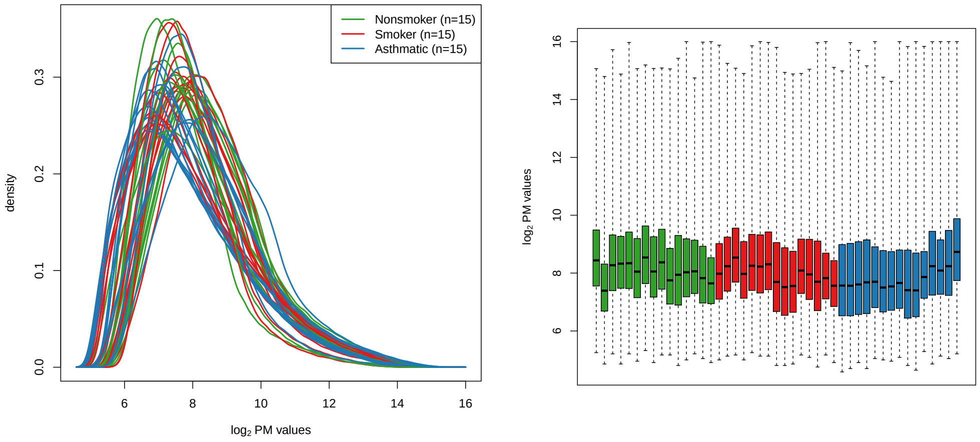

Figure 3: Densities and box plots of the raw PM values using the $n = 45$ Affymetrix Human Gene Chip arrays in the *alveolarSmokingAffyData* data from [5]. The samples are colored by disease status: Nonsmoking (green), Smoking (red), Asthma (blue). Using `quantro`, we report there are no global differences detected at the $\alpha = 0.05$ level between the distributions across the three groups ($p = 0.562$).

lungCOPDAffyData. We examined 238 Affymetrix Human Gene 1.0 ST arrays (GEO accession GSE37147) [6] which compared the gene expression of bronchial brushings from $n = 87$ samples with chronic obstructive pulmonary disease (COPD) and $n = 151$ samples without COPD. The densities and box plots of the raw PM values are shown in Figure 4 and colored by disease status: No COPD (green), COPD (red). We tested for global differences in the distributions between the two groups using `quantro`. We assessed the statistical significance of the test statistic ($F_{quantro} = 1.45$) using permutation testing and report there were no global differences detected at the $\alpha = 0.05$ level between the groups ($p = 0.218$).

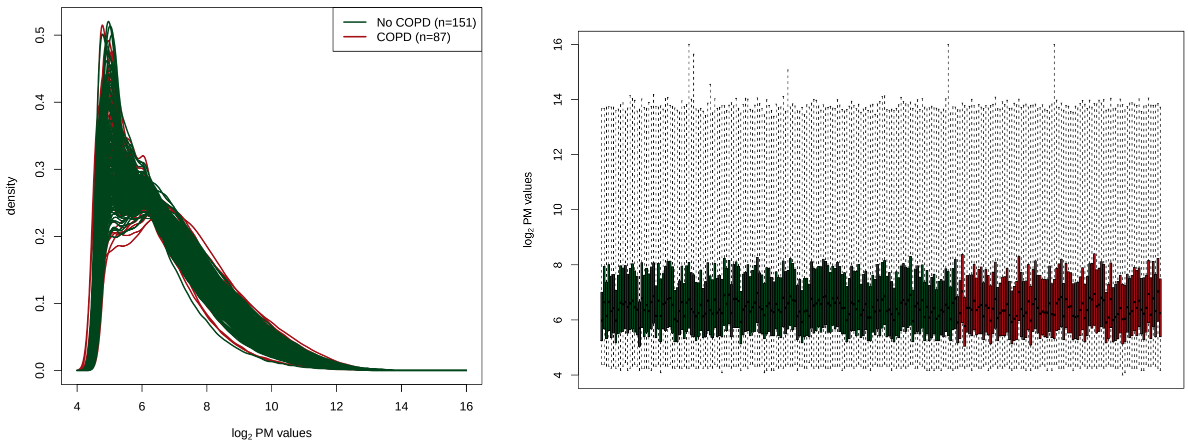

Figure 4: Densities and box plots of the raw PM values using the $n = 238$ Affymetrix Human Gene 1.0 ST arrays in the *lungCOPDAffyData* data from [6]. The samples are colored by disease status: No COPD (green) and COPD (red). Using `quantro`, we report there are no global differences detected at the $\alpha = 0.05$ level between the distributions of the No COPD and COPD samples ($p = 0.218$).

brainParkinsonsAffyData. We examined 22 Affymetrix arrays (GEO accession GSE19587) [7] which compared gene expression from two regions in the brain using control patients ($n = 10$) and patients diagnosed with Parkinson's disease ($n = 12$). The two regions of the brain compared were Dorsal Motor Nucleus of the Vagus (DMNV) and Inferior Olivary Nucleus (ION). The densities and box plots of the raw PM values are shown in Figure 5 and colored by region in the brain: DMNV (green) and ION (red). We tested for global differences in the distributions between the two regions of the brain in each set of samples using `quantro`. We assessed the statistical significance of the test statistic ($F_{quantro} = 3.097$ (only control samples), $F_{quantro} = 1.441$ (only Parkinson's samples)) using permutation testing. We report there were no global differences detected at the $\alpha = 0.05$ level between the distributions in both the control samples ($p = 0.119$) and in the Parkinson's samples ($p = 0.264$). We note there is a large amount of variation within each group (both in the controls and in the Parkinson's samples). Therefore, `quantro` has a limited amount of power to detect any global differences because the variation is so high within each group and the sample size is small for each group.

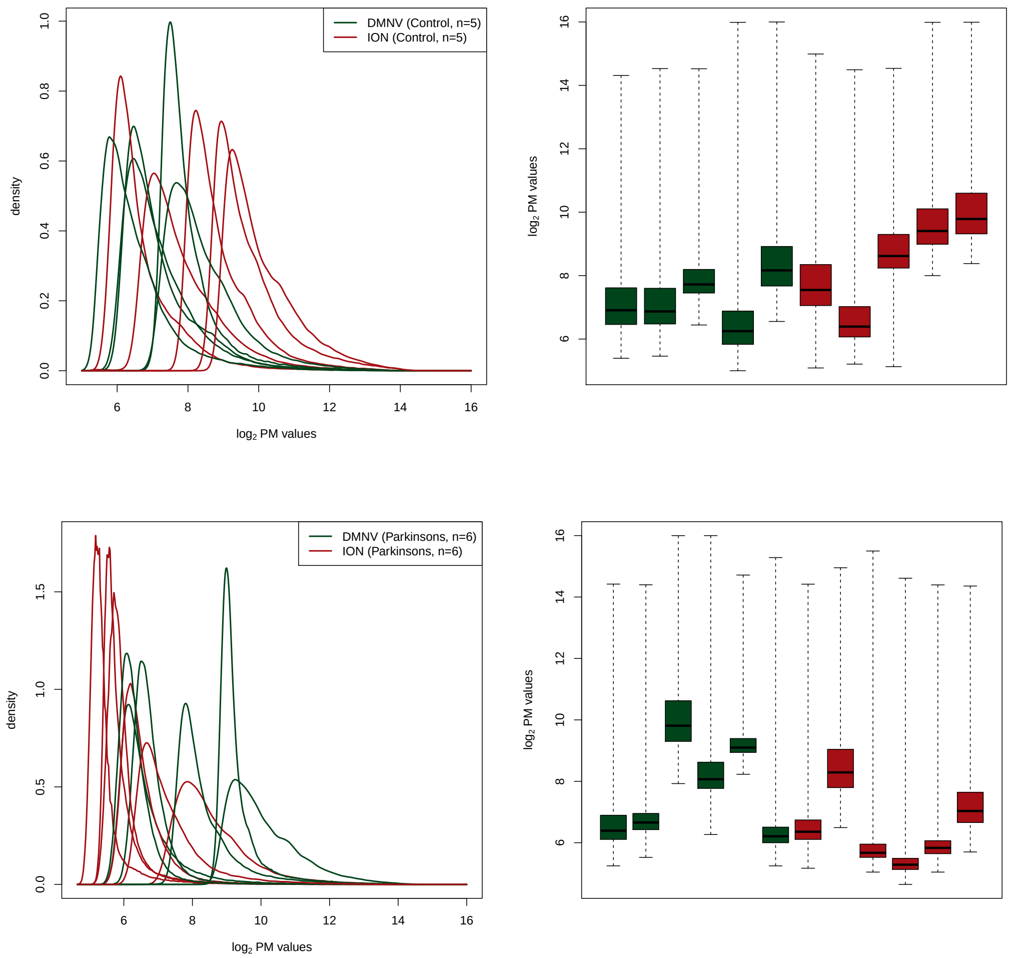

Figure 5: Densities and box plots of the raw PM values using control samples (top row) and Parkinsons's samples (bottom row) from the $n = 22$ Affymetrix GeneChip arrays in [7]. The samples are colored by region in the brain: DMNV (green) and ION (red). Using `quantro`, we report there are no global differences detected at the $\alpha = 0.05$ level between the distributions of the DMNV and ION samples in both the control samples ($p = 0.119$) and in the Parkinson's samples ($p = 0.264$).

brainLiverAffyData. We extracted 82 Affymetrix samples from brain tissues ($n = 42$) and liver tissues ($n = 40$). These samples were from multiple GEO data sets with the largest number of normal brain samples (GSE17612, GSE21935) and normal liver samples (GSE14668, GSE29721, GSE38941), respectively. We investigated if there were global differences in the distributions between the two tissues. The densities and box plots of the raw PM values are shown in Figure 6 and colored by tissue: brain (green) and liver (red). The shades of green and red represent the different GEO data sets (i.e. different batches) within each tissue. We tested for global differences in the distributions between the tissues using `quantro`. We assessed the statistical significance of the test statistic ($F_{quantro} = 7.373$) using permutation testing and report there are global differences between the distributions of the brain and liver tissues ($p = 0.004$).

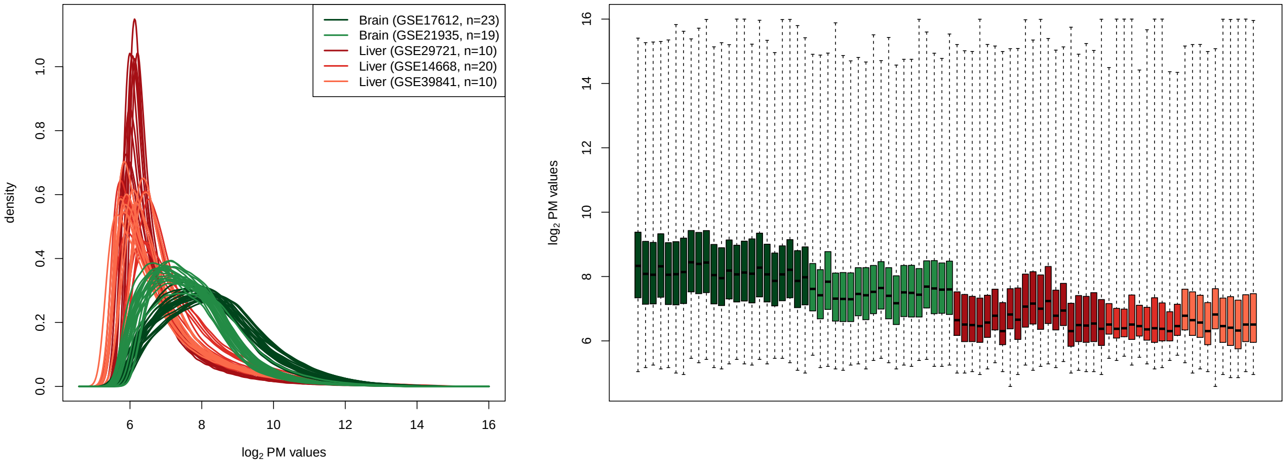

Figure 6: Densities and box plots of the raw PM values from the $n = 82$ Affymetrix GeneChip arrays in brainLiverAffyData. The samples are colored by tissue: brain (green) and liver (red). Using `quantro`, we report there are global differences between the distributions of the brain and liver tissues ($p = 0.004$).

lungCancerAffyData. We extracted 444 Affymetrix lung samples representing normal ($n = 170$) and tumor ($n = 274$) samples. These samples were from GEO data sets with the largest number of normal lung samples (GSE18842, GSE19188, GSE19804) and tumor lung samples (GSE10445, GSE12667, GSE2109). We investigated if there were global differences in the distributions between the normal and tumor samples. The densities and box plots of the raw PM values are shown in Figures 7 and colored by tumor status: normal (green) and tumor (red). The shades of green and red represent the different GEO data sets (i.e. different batches) within each tumor status. We tested for global differences in the distributions between the groups using `quantro`. We assessed the statistical significance of the test statistic ($F_{quantro} = 110.10$) using permutation testing and report there are global differences between the distributions of the normal and tumor lung samples ($p < 0.001$).

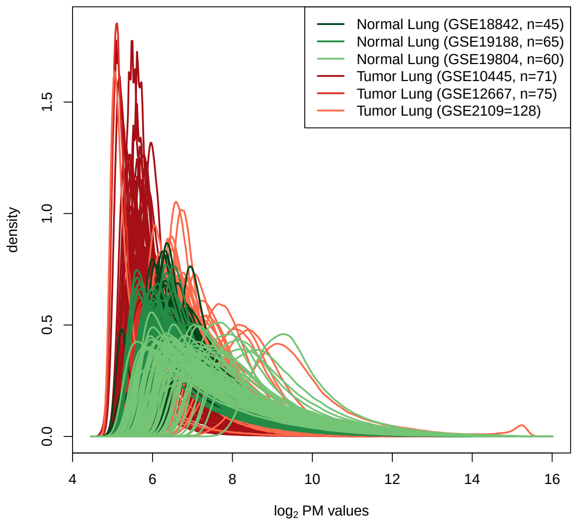

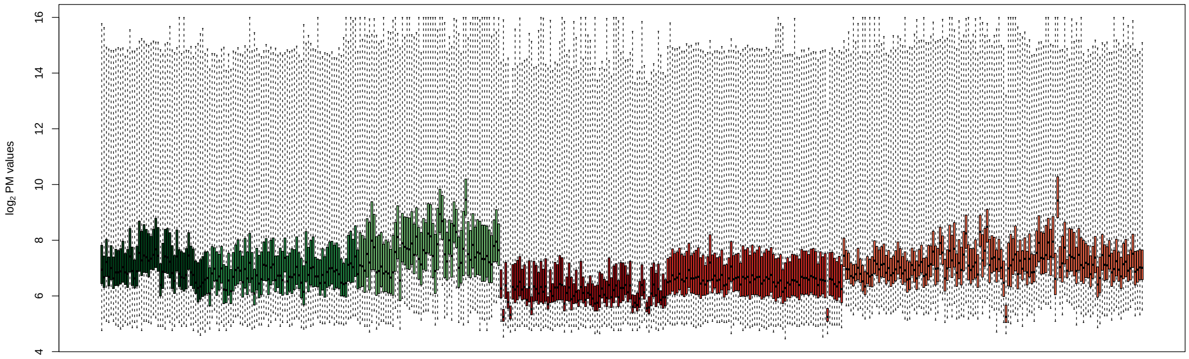

Figure 7: Densities and boxplots of the raw PM values from the $n = 444$ Affymetrix GeneChip arrays in *lungCancerAffyData*. The samples are colored by tumor status: normal (green) and tumor (red). Using `quantro`, we report there are global differences between the distributions of the normal and tumor lung samples ($p < 0.001$).

breastCancerAffyData. We extracted 931 Affymetrix breast samples representing normal ($n = 289$) and tumor ($n = 674$) samples. These samples were from GEO data sets with the largest number of normal breast samples (GSE10780, GSE10810, GSE29431, GSE30010) and tumor breast samples (GSE2109, GSE5460, GSE653, GSE9195). We investigated if there were global differences in the distributions between the normal and tumor samples. The densities of the raw PM values are shown in Figure 8 and colored by tumor status: normal (green) and tumor (red). The shades of green and red represent the different GEO data sets (i.e. different batches) within each tumor status. We tested for global differences in the distributions between the groups using `quantro`. We assessed the statistical significance of the test statistic ($F_{quantro} = 308.8$) using permutation testing and report there are global differences between the distributions of the normal and tumor breast samples ($p < 0.001$).

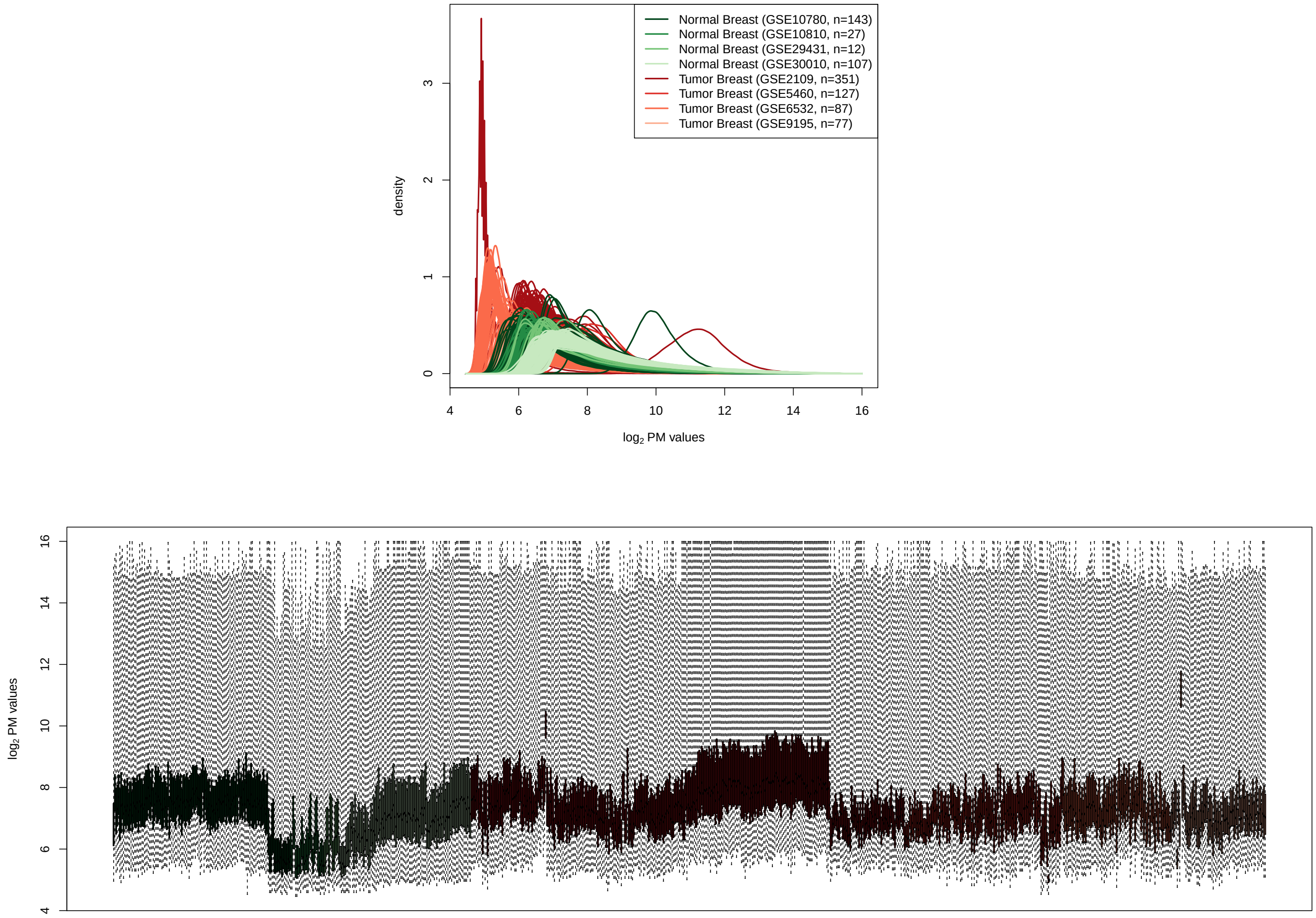

Figure 8: Densities and boxplots of the raw PM values from the $n = 931$ Affymetrix GeneChip arrays in *breastCancerAffyData*. The samples are colored by tumor status: normal (green) and tumor (red). Using `quantro`, we report there are global differences between the distributions of the normal and tumor breast samples ($p < 0.001$).

prostateCancerAffyData. We extracted 167 Affymetrix prostate samples representing normal ($n = 85$) and tumor ($n = 82$) samples. These samples were from GEO data sets with the largest number of normal prostate samples (GSE17951, GSE32448) and tumor prostate samples (GSE2109). We investigated if there were global differences in the distributions between the normal and tumor samples. The densities and box plots of the raw PM values are shown in Figure 9 and colored by tumor status: normal (green) and tumor (red). The shades of green and red represent the different GEO data sets (i.e. different batches) within each tumor status. We tested for global differences in the distributions between the groups using `quantro`. We assessed the statistical significance of the test statistic ($F_{quantro} = 16.95$) using permutation testing and report there are global differences between the distributions of the normal and tumor prostate samples ($p < 0.001$).

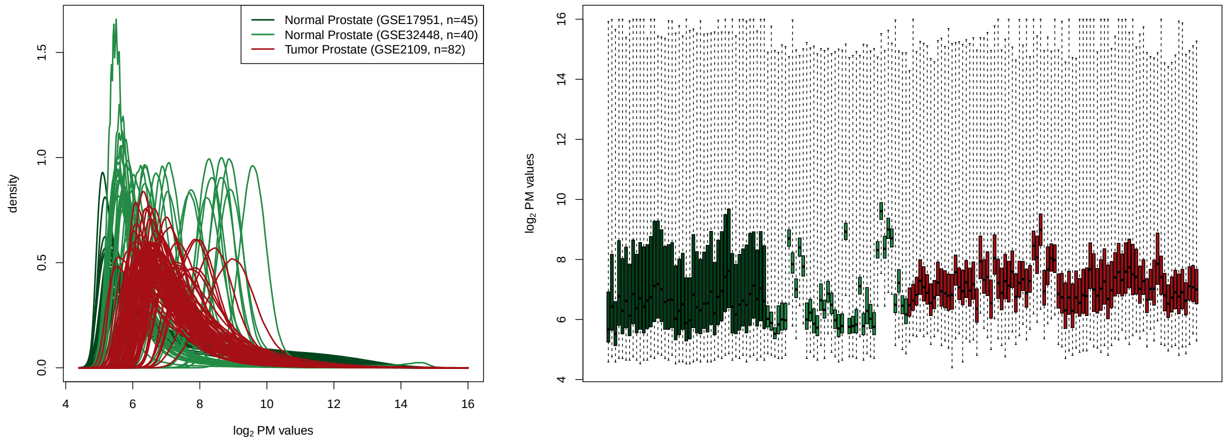

Figure 9: Densities and box plots of the raw PM values from the $n = 167$ Affymetrix GeneChip arrays in *prostateCancerAffyData*. The samples are colored by tumor status: normal (green) and tumor (red). Using `quantro`, we report there are global differences between the distributions of the normal and tumor prostate samples ($p < 0.001$).

thyroidCancerAffyData. We extracted 98 Affymetrix thyroid samples representing normal ($n = 65$) and tumor ($n = 33$) samples. These samples were from GEO data sets with the largest number of normal thyroid samples (GSE29265, GSE33630) and tumor thyroid samples (GSE2109). We investigated if there were global differences in the distributions between the normal and tumor samples. The densities and box plots of the raw PM values are shown in Figure 10 and colored by tumor status: normal (green) and tumor (red). The shades of green and red represent the different GEO data sets (i.e. different batches) within each tumor status. We tested for global differences in the distributions between the groups using `quantro`. We assessed the statistical significance of the test statistic ($F_{quantro} = 19.94$) using permutation testing report there are global differences between the distributions of the normal and tumor thyroid samples ($p < 0.001$).

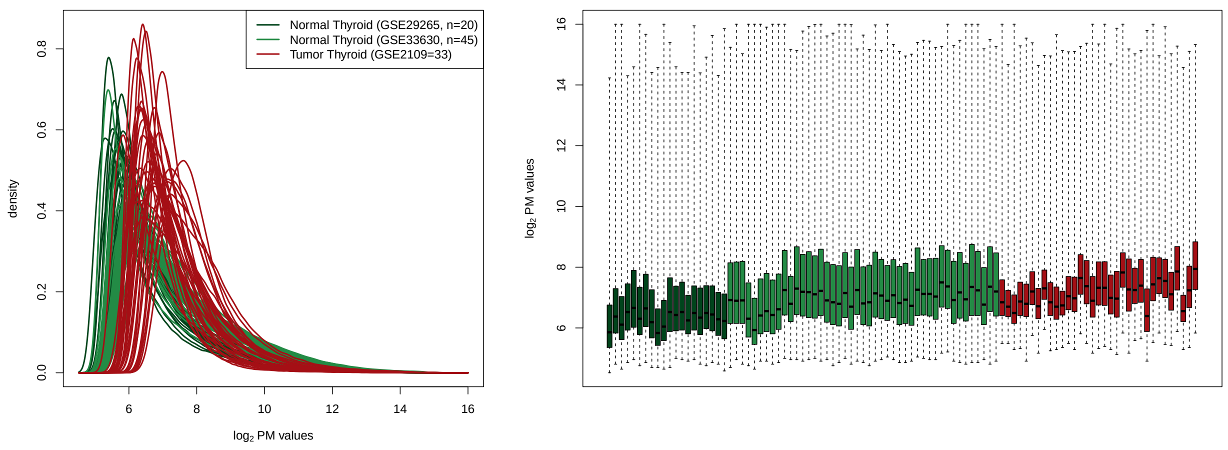

Figure 10: Densities and box plots of the raw PM values from the $n = 98$ Affymetrix GeneChip arrays in *thyroidCancerAffyData*. The samples are colored by tumor status: normal (green) and tumor (red). Using `quantro`, we report there are global differences between the distributions of the normal and tumor thyroid samples ($p < 0.001$).

stomachCancerAffyData. We investigated 82 Affymetrix stomach samples representing normal ($n = 31$) and tumor ($n = 51$) samples. These samples were from GEO data sets with the largest number of normal stomach samples (GSE13911) and tumor stomach samples (GSE13911, GSE2109). We investigated if there were global differences in the distributions between the normal and tumor samples. The densities and box plots of the raw PM values are shown in Figure 11 and colored by tumor status: normal (green) and tumor (red). The shades of green and red represent the different GEO data sets (i.e. different batches) within each tumor status. We tested for global differences in the distributions between the groups using `quantro`. We assessed the statistical significance of the test statistic ($F_{quantro} = 11.81$) using permutation testing and report there are global differences between the distributions of the normal and tumor stomach samples ($p < 0.001$). Because there were both normal and tumor stomach samples within the same GEO data set (GSE13911), we tested for global differences using only these n = 69 samples in Figure 11. Again, we report global differences ($F_{quantro} = 11.46$) between the normal and tumor stomach samples ($p < 0.001$).

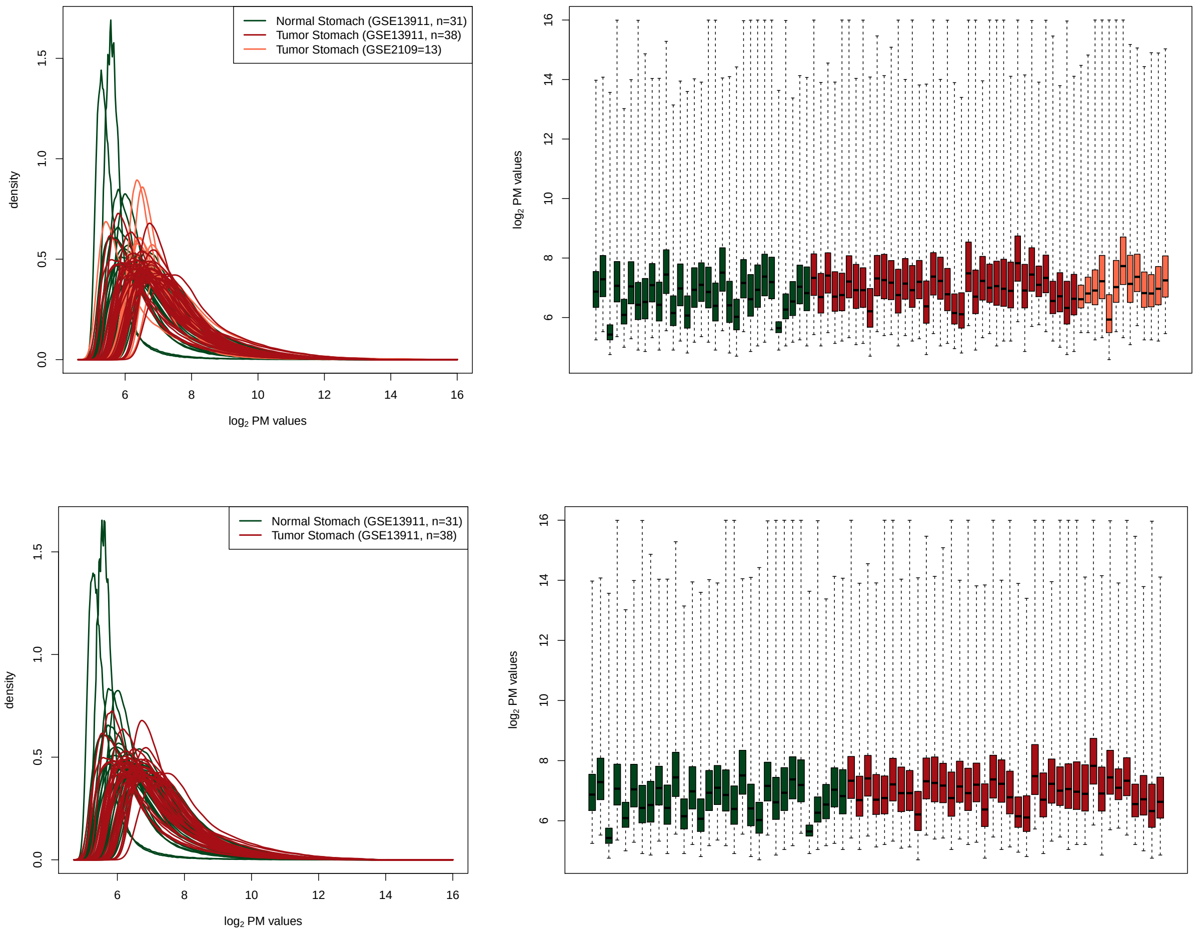

Figure 11: Densities and box plots of the raw PM values from the $n = 82$ (top row) and $n = 69$ (bottom row) Affymetrix GeneChip arrays in *stomachCancerAffyData*. The $n = 69$ samples are a subset of the samples from the original $n = 82$, but these samples are all from the same GEO data set (GSE13911). The samples are colored by tumor status: normal (green) and tumor (red). Using `quantro`, we report there are global differences between the distributions of the $n = 82$ normal and tumor stomach samples ($p < 0.001$) and the $n = 69$ normal and tumor stomach samples ($p < 0.001$).

liverCancerAffyData. We extracted 167 Affymetrix liver samples representing normal ($n = 40$) and tumor ($n = 136$) samples. These samples were from GEO data sets with the largest number of normal liver samples (GSE14668, GSE29721, GSE38941) and tumor liver samples (GSE2109, GSE9829;GSE9843). We investigated if there were global differences in the distributions between the normal and tumor samples. The densities and box plots of the raw PM values are shown in Figure 12 and colored by tumor status: normal (green) and tumor (red). The shades of green and red represent the different GEO data sets (i.e. different batches) within each tumor status. We tested for global differences in the distributions between the groups using `quantro`. We assessed the statistical significance of the test statistic ($F_{quantro} = 3.502$) using permutation testing and report there are global differences detected at the $\alpha = 0.05$ level between the distributions of the normal and tumor liver samples ($p = 0.044$).

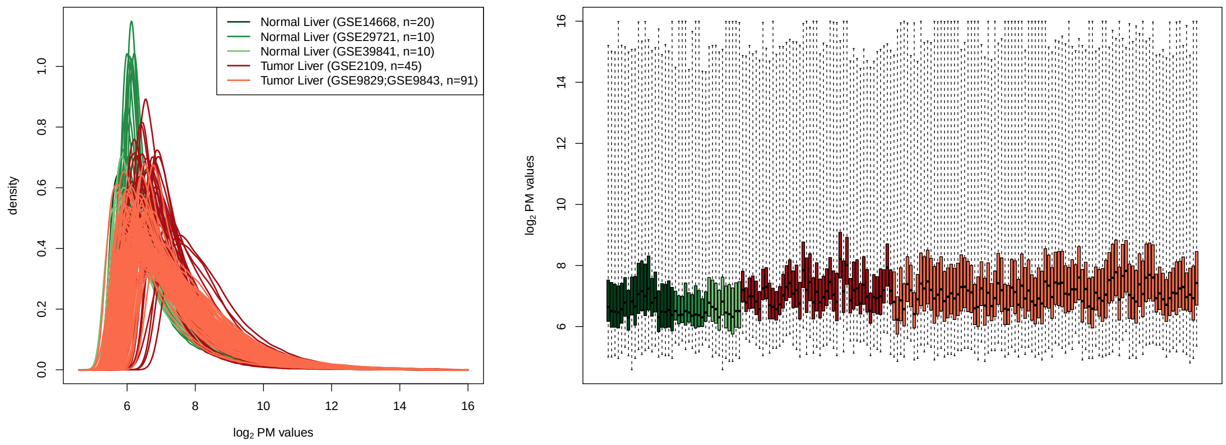

Figure 12: Densities and box plots of the raw PM values from the $n = 167$ Affymetrix GeneChip arrays in *liverCancerAffyData*. The samples are colored by tumor status: normal (green) and tumor (red). Using `quantro`, there are global differences detected at the $\alpha = 0.05$ level between the distributions of the normal and tumor liver samples ($p = 0.044$).

liverNAFLDAffyData. We examined 73 Affymetrix arrays (GEO accession GSE48452) [8] which compared the gene expression of liver tissues grouped into $n = 14$ control, $n = 27$ healthy obese, $n = 14$ steatosis, and $n = 18$ nash samples. The densities and box plots of the raw PM values are shown in Figure 13 and colored by disease status: Control (orange), Healthy obese (red), Steatosis (green), Nash (blue). We tested for global differences in the distributions between the two groups using `quantro`. We assessed the statistical significance of the test statistic ($F_{quantro} = 4.286$) using permutation testing and report there are global differences between the distributions of the NAFLD samples ($p = 0.004$).

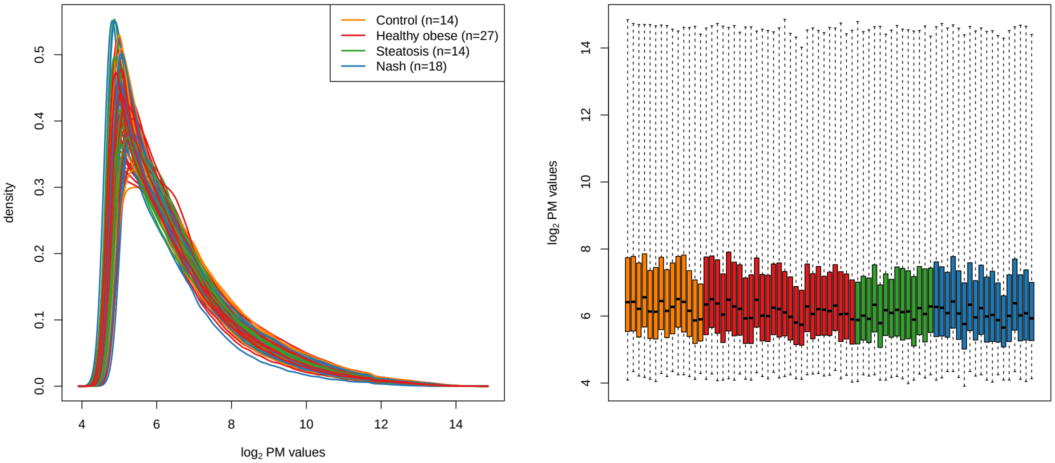

Figure 13: Densities and box plots of the raw PM values using the $n = 73$ Affymetrix arrays in the *liverNAFLDAffy-Data* data from [8]. The samples are colored by disease status: Control (orange), Healthy obese (red), Steatosis (green), Nash (blue). Using `quantro`, we report there are global differences between the distributions of NAFLD samples ($p = 0.004$).

2.2 DNA methylation

2.2.1 Microarrays

We used the *minfi* R/Bioconductor package [15] to analyze Illumina Infinium 450K arrays. We extracted the raw methylated and unmethylated signal using the `preprocessRaw()` function the *cellcompMethyl* data set. The raw methylated and unmethylated counts were provided as text files for the *pancreaticT2DMethyl* and *adiposeExerciseMethyl* data sets (http://www.ludc.med.lu.se/research-units/epigenetics-and-diabetes/published-data). The "beta"-values were computed from the methylated and unmethylated counts using the `getBeta()` function using Illumina's formula with an offset = 100.

$$\beta = \frac{M}{M + U + 100}$$

adiposeExerciseMethyl. We examined the DNA methylation of 46 adipose tissue samples comparing men before and after six months of exercise [10]. This study was performed to find differentially methylated CpGs between healthy men before ($n = 23$) and after ($n = 23$) six months of exercise on the Illumina Infinium HumanMethylation450 BeadChip array. For this analysis, we used the raw beta values to test for global differences in the distributions between the two groups: before exercise, after exercise. The densities and box plots of the raw beta values are shown in Figure 14 and colored by disease status: before 6 months of exercise (green) and after 6 months of exercise (red). We tested for global differences in the distributions between the groups using `quantro`. We assessed the statistical significance of the test statistic ($F_{quantro} = 2.092$) using permutation testing and report there were no global differences detected at the $\alpha = 0.05$ level between the distributions of healthy men before and after six months of exercise ($p = 0.132$).

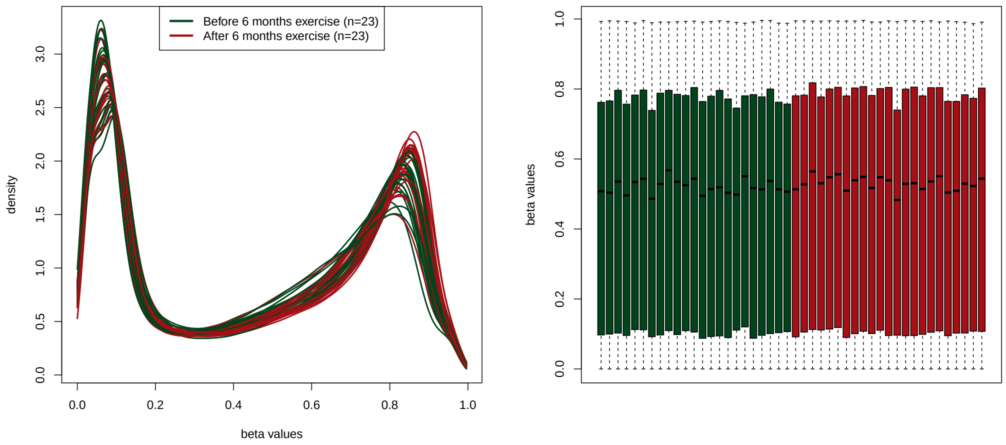

Figure 14: Densities and box plots of the raw beta values from the $n = 46$ Illumina Infinium HumanMethylation450 BeadChip arrays in the *adiposeExerciseMethyl* data from [10]. The samples are colored by disease status: before (green) and after six months of exercise (red). Using `quantro`, we report there are no global differences detected at the $\alpha = 0.05$ level between the distributions using the raw beta values of healthy men before and after six months of exercise ($p = 0.132$).

pancreaticT2DMethyl. We examined the DNA methylation of 49 pancreatic tissue samples from non-diabetic and type 2 diabetes (T2D) patients [11]. This study was performed to find differentially methylated CpGs in T2D patients compared to donors not diagnosed with diabetes using the Illumina Infinium HumanMethylation450 BeadChip array. The authors analyzed $n = 15$ T2D samples and $n = 34$ non-diabetic samples. For this analysis, we used the raw beta values to test for global differences in the distributions between the two groups: non-diabetic and T2D. The densities and box plots of the raw beta values are shown in Figure 15 and colored by disease status: non-diabetic (green) and T2D (red). We tested for global differences in the distributions between the groups using `quantro`. We assessed the statistical significance of the test statistic ($F_{quantro} = 2.854$) using permutation testing and report there were no global differences detected at the $\alpha = 0.05$ level between the distributions of the non-diabetic and T2D samples ($p = 0.069$).

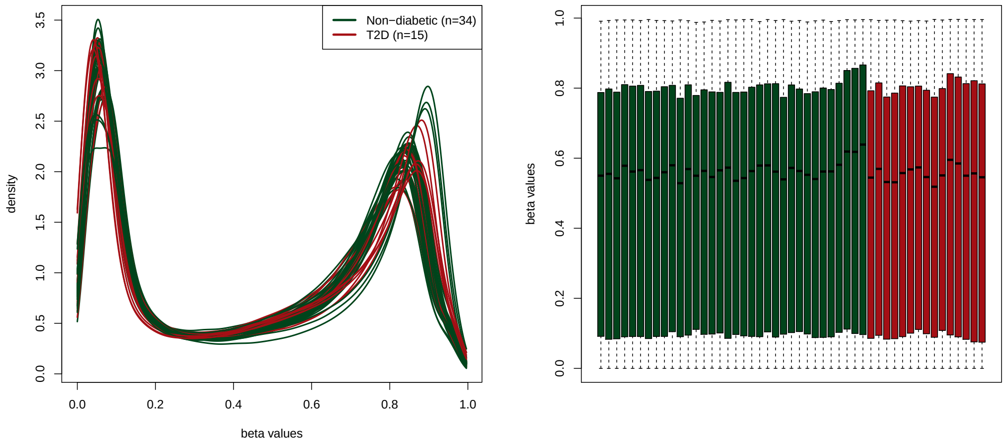

Figure 15: Densities and box plots of the raw beta values from the $n = 49$ Illumina Infinium HumanMethylation450 BeadChip arrays in the *pancreaticT2DMethyl* data from [11]. The samples are colored by disease status: non-diabetic (green) and T2D (red). Using `quantro`, we report there were no global differences detected at the $\alpha = 0.05$ level between the distributions of the non-diabetic and T2D samples ($p = 0.069$).

cellcompMethyl. We examined the DNA methylation of 36 samples of purified cell types from whole blood [12]. This study was originally performed to determine if whole blood is a valid source for DNA methylation analysis using the Illumina Infinium HumanMethylation450 BeadChip array. The authors analyzed 60 samples (10 cell populations from 6 healthy male blood donors ages 38 ± 13.6 years) to compare cell populations and identify differentially methylated CpG sites unique to each cell type. For this analysis, we used the raw beta values to test for global differences in the distributions across purified cell types. The 6 purified cell types were CD14+ Monocytes (Mono), CD19+ B-cells (Bcell), CD4+ T-cells (CD4T), CD56+ NK-cells (NK), CD8+ T-cells (CD8T), and Granulocytes (Gran). The sample 'CD8+ 105' was identified as an outlier sample and removed from this analysis. The densities and box plots of the beta values are shown in Figure 16 and colored by cell type. We tested for global differences in the distributions between the groups using `quantro`. We assessed the statistical significance of the test statistic ($F_{quantro} = 6.797$) using permutation testing and report there are global differences between the distributions of purified cell types ($p < 0.001$).

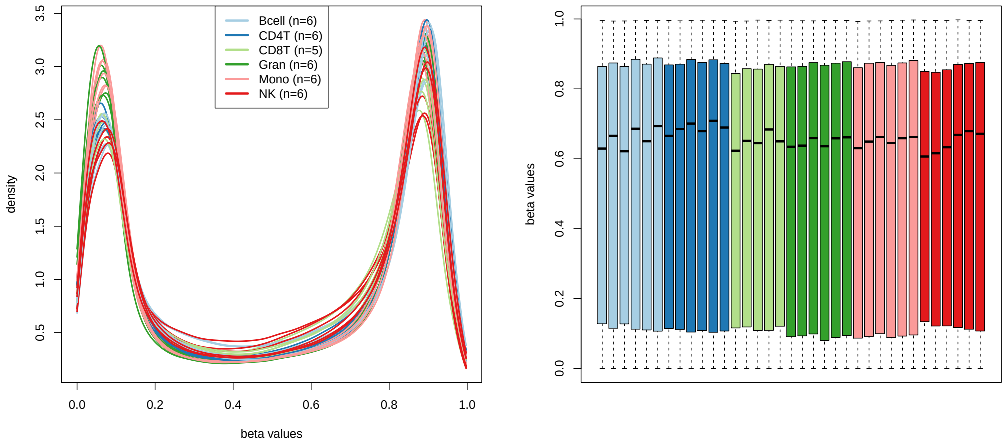

Figure 16: Densities and box plots of the raw beta values from the $n = 35$ Illumina Infinium HumanMethylation450 BeadChip arrays in the *cellcompMethyl* data across $n = 6$ purified cell types [12]. The samples are colored by cell type. Using `quantro`, we report there are global differences between the distributions across the six cell types using the raw beta values ($p < 0.001$).

3 `quantroSim`: An R-package to simulate gene expression and DNA methylation data

To evaluate the performance of `quantro` and quantile normalization, we developed an R-package, referred to as `quantroSim`, to simulate gene expression and DNA methylation data. The models to simulate gene expression (and DNA methylation data) start by defining true biological differences (unobserved) between a set of groups that is not based on using any platform technology. Next, we define a set of platform-specific parameters that create observed technical variability and simulate a set of samples from each group. Our model controls the proportion of true differences between groups and controls of the magnitude of the technical variation from the platform technology. To simulate gene expression and DNA methylation samples using a microarray platform technology, we use the Langmuir adsorption model [16] to model the background noise of non-specific binding, the optical noise from the florescence intensity of the scanner and the chemical saturation in the hybridization of the probes.

3.1 Simulating gene expression samples

To simulate the real gene expression in a given sample, we start with gene-level information. If at least one copy of a given RNA molecule exists in the sample, then that molecule transcribed from a given gene is being expressed. Let n_g = the number of RNA molecules expressed from g^{th} gene where $g \in (1, \ldots, G)$. We assume n_g follows a zero-inflated Poisson distribution:

$$\begin{aligned} P(n_g = 0) &= \pi + (1-\pi)e^{-\lambda_g} \\ P(n_g = c) &= (1-\pi)\frac{(\lambda_g)^c e^{-\lambda_g}}{c!} \qquad \text{where } c \geq 1 \end{aligned}$$

where π is the parameter representing the inflated proportion of zeros and $\log_2(\lambda_g) \sim N(\mu_g, \sigma_g^2)$.

To compare the gene expression between $K = 2$ groups, we define n_{gk} as the number of RNA molecules expressed from the gth gene in the kth group. We define `pDiff` as the proportion of genes that have increased gene expression between the first group ($k = 1$) and the second group ($k = 2$). Define `D` as the set genes that are different between the first group and the second group. We define n_{gk} in the following way:

$$n_{gk} = \begin{cases} n_g & \text{if } k = 1, \forall g \in (1, \ldots G) \\ n_g & \text{if } k = 2, g \notin \mathtt{D} \\ n_g * \gamma_g & \text{if } k = 2, g \in \mathtt{D} \end{cases}$$

where γ_g is a fold change (e.g. $\gamma_g = 5$).

3.1.1 Microarrays

In Affymetrix arrays each gene is represented by 11-20 probes pairs: perfect matches (PM) and mismatches (MM). For our purposes, we only simulate PM probes. To simulate probe-level fragments, let N_g = the number of probes for the gth gene

$$N_g \sim Binomial(20, 0.6)$$

Each RNA molecule from the gth gene in the kth group is sheared into fragments and $\boldsymbol{z}_{gk}^T = (z_{1gk}, \ldots, z_{N_g,gk})$ are the number fragments in the sample represented by the N_g probes. If there are n_{gk} RNA molecules, then $z_{jgk} \leq n_{gk}$. Let p_{jg} be the probability of successfully creating a matching fragment for the jth probe from the n_{gk} RNA molecules. We assume $p_{jg} \sim Unif(0, 1)$, then we can simulate

$$z_{jgk} \sim Binomial(n_{gk}, p_{jg})$$

PCR amplification is sometimes used to amplify the RNA. This is not a requirement for the simulations, but the option is available. To simulate PCR, we use probability generating functions [17]. Let q_{jg} = probability that the RNA fragment in the jth probe in g^{th} gene replicates itself during one PCR cycle. If there are z_{jgk} RNA molecules before PCR, then the expected number of RNA molecules after N_{PCR} PCR cycles is

$$x_{jgk} = z_{jgk}(1 + q_{jg})^{N_{PCR}}$$

where $q_{jg} \sim Unif(0, 1)$. If PCR is not used in the simulation then, $x_{ijk} = z_{ijk}$. To keep the notation simple, we drop the gth gene notation and consider just x_{jk}. To model the saturation probe effect in microarrays, we use the Langmuir adsorption model [16]. The "intensity" of the PM probes can be modeled using

$$PM_{ijk} = o_{ijk} + d_{ijk} + a_{ijk}(\frac{x_{jk}}{x_{jk} + b_{ijk}})\epsilon_{ijk}$$

where $i \in (1, \ldots, n_k)$ represents the sample, $j \in (1, \ldots, J)$ represents the probe and $k \in (1, \ldots, K)$ represents the group index. Let T = total number of samples.

We define the optical noise (o_{ijk}) to be written as a product of sample-level optical noise (o_{ik}) and probe-level optical noise (o_j) or $o_{ijk} = o_{ik} * o_j$ to allow for global shifts between samples. Both optical noise parameters are simulated using a lognormal normal distribution. Similarly, the florescence intensity from the scanner ($a_{ijk} = a_{ik} * a_j$) and the parameter ($b_{ijk} = b_{ik} * b_j$) are also a product of sample-level parameter and probe-level parameter to allow for global scaling between samples. The background noise (d_{ijk}), measurement error (ϵ_{ijk}) are also simulated using a lognormal distribution. Table 2 contains a list of the example parameters used to simulate gene expression samples using microarrays. Each of the parameters sample-level noise (a_{ik}) and probe-level noise parameters (a_j) have their

own hyperparameters to allow for global shifts. Similarly, $b_{ijk} = b_{ik} * b_j$ and $o_{ijk} = o_{ik} * o_j$ (optical noise) with their own set of hyperparameters.

Using the multivariate normal distribution, we simulate $\log_2(a)$, $\log_2(b)$ and $\log_2(o)$ with a given set of hyperparameters (see Table 2). In the `quantroSim` R-package, the variance hyperparameters are referred to as `siga`, `sigb` and `sigOpt` in the `simulateGEx()` function. In `quantroSim`, the level of technical variation induced from the platform technology is controlled using the covariance matricies of `siga`, `sigb` and `sigOpt` in the function `simulateGEx()`. There is a vignette available in the `quantroSim` R-package that contains more details about the `simulateGEx()` function. Here we define "low" technical variation as the default parameters in the functions `simulateGEx()` (Table 2) and "high" technical variation as ten fold increase. An example of simulated gene expression arrays with "low" and "high" technical variation and default parameters otherwise is given in Figure 17 and 18, respectively.

```
library(quantroSim)
set.seed(999)
siga = sigb = sigOpt = 0.1 * diag(10)
geneTruth <- simulateGExTruth(nGenes = 2e4, nGroups = 2, pDiff = 0.05,
                              foldDiff = 5, verbose = FALSE)
sim <- simulateGEx(geneTruth, GEx.platform = "GExArrays", nSamps = 5,
                   siga = siga, sigb = sigb, sigOpt = sigOpt, verbose = FALSE)
plotGEx(sim)
```

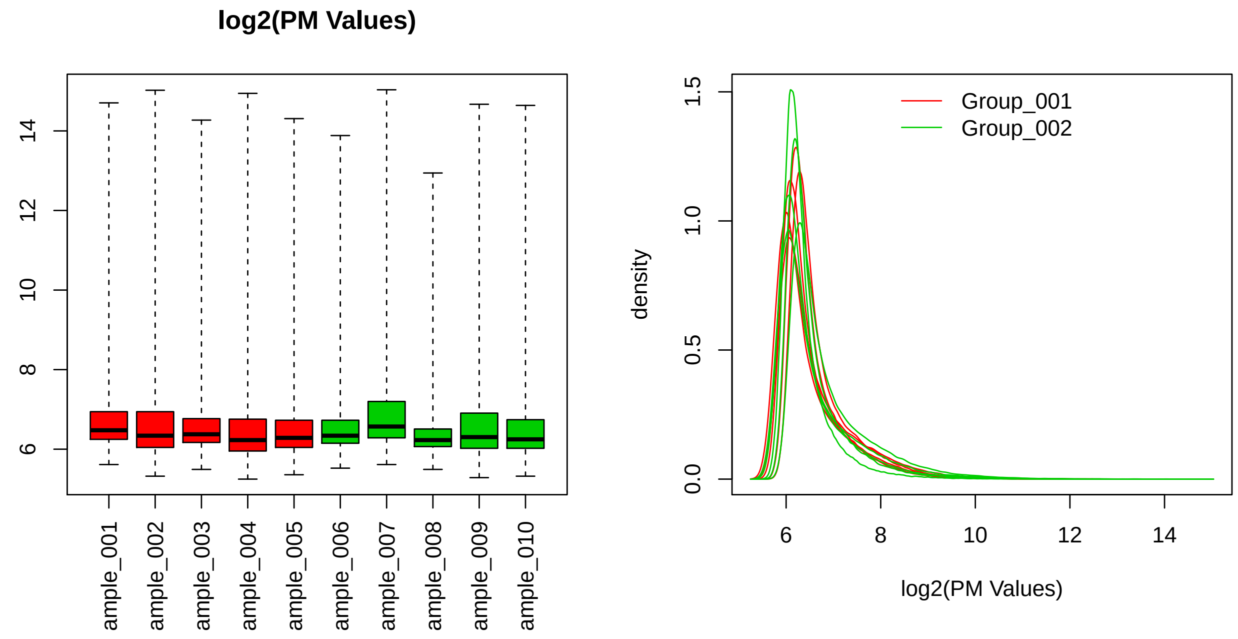

Figure 17: An example using the `simulateGExTruth()` and `simulateGEx()` functions in the `quantroSim` package to simulate 10 gene expression arrays each with `nGenes` = 20000 genes and 5% of the genes differentially expressed between two groups (5 samples in each group). The differentially expressed genes were simulated with a five fold increase in expression. The default parameters in `simulateGEx()` are used for the level of technical variability.

```
set.seed(999)
siga = sigb = sigOpt = 1 * diag(10)
geneTruth <- simulateGExTruth(nGenes = 2e4, nGroups = 2, pDiff = 0.05,
                              foldDiff = 5, verbose = FALSE)
sim <- simulateGEx(geneTruth, GEx.platform = "GExArrays", nSamps = 5,
                   siga = siga, sigb = sigb, sigOpt = sigOpt, verbose = FALSE)
plotGEx(sim)
```

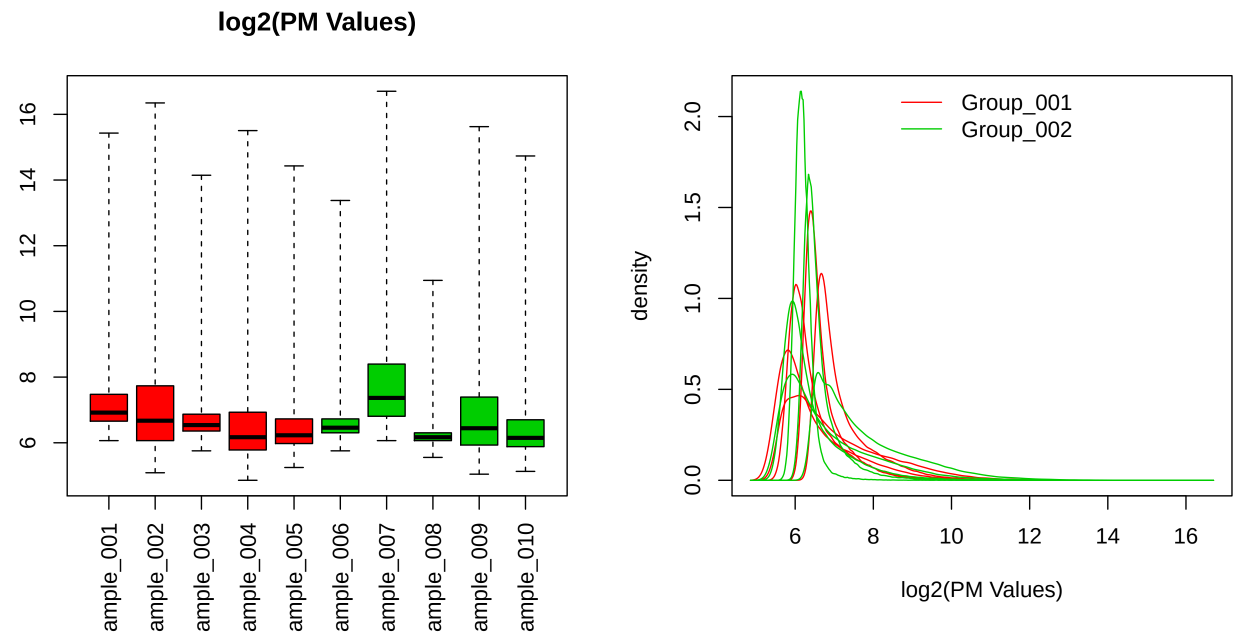

Figure 18: An example using the `simulateGExTruth()` and `simulateGEx()` functions in the `quantroSim` package to simulate 10 gene expression arrays each with `nGenes` = 20000 genes and 5% of the genes differentially expressed between two groups (5 samples in each group). The differentially expressed genes were simulated with a five fold increase in expression. The parameters of `siga`, `sigb` and `sigOpt` were increased to increase the level of technical variability.

3.2 Simulating DNA methylation samples

To simulate real (unobservable) DNA methylation in a given sample, we start with CpG-level information. A given CpG can be considered to be unmethylated (state 0), semi-methylated (state 1), methylated (state 2). Using these three states, we define θ_j as the true proportion of methylation at the jth CpG site where $j \in (1, \ldots, J)$ and

$$\theta_j = \frac{1}{1 + \exp(z_j)}$$

and z_j follows a mixture of three normal distributions

$$z_j \sim \pi_0 N(\mu_0, \sigma_0^2) + \pi_1 N(\mu_1, \sigma_1^2) + (1 - \pi_0 - \pi_1) N(\mu_2, \sigma_2^2)$$

and $S_j \in \{0, 1, 2\}$ represents the true state of methylation for the jth CpG.

Table 2: Default parameters for distributions to simulate gene expression samples using microarrays.

	Parameter	Distribution
	$\log_2(o_{ik})$	$N(5, 0.1)$
sample-level	$\log_2(a_{ik})$	$N(20, 0.1)$
	$\log_2(b_{ik})$	$N(18, 0.1)$
	$\log_2(o_j)$	$N(0, 0.01)$
	$\log_2(a_j)$	$N(0, 0.01)$
probe-level	$\log_2(b_j)$	$N(0, 0.01)$
	$\log_2(d_j)$	$N(5, 0.1)$
	$\log_2(\epsilon_j)$	$N(0, 0.1)$

To compare the DNA methylation between $k = 2$ groups, we define θ_{jk} as the true proportion of methylation at the jth CpG site in the kth group. We define `pDiff` as the proportion of CpG sites that are different between the first group ($k = 1$) and the second group ($k = 2$) and `pUp` as the proportion of `pDiff` CpGs that change from an unmethylated ($S_j = 0$) to a semi-methylated or a methylated ($S_j = \{1, 2\}$) state. If we start with $J = 1000$ CpGs and initially 40% are in state $S_j = 0$ and 60% are in state $S_j = \{1, 2\}$, then there can be at most 400 CpGs that move from $S_j = 0$ to $S_j = \{1, 2\}$, or

$$\min(1000 * \texttt{pDiff} * \texttt{pUp}, 400)$$

Similarly, there are (1 - `pUp`) CpGs of the `pDiff` CpGs that will change from a semi-methylated or a methylated ($S_j = \{1, 2\}$) to an unmethylated ($S_j = 0$) state. Continuing with the example above, if we start with $J = 1000$ CpGs and intially 40% are in state $S_j = 0$ and 60% are in state $S_j = \{1, 2\}$, then there can be at most 600 CpGs that move from $S_j = \{1, 2\}$ to $S_j = 0$, or

$$\min(1000 * \texttt{pDiff} * (1 - \texttt{pUp}), 600)$$

Now, define `D` as the set CpG sites that are different between the first group and the second group. We simulate z_{jk} in the following way:

$$z_{jk} = \begin{cases} z_j + \epsilon & \text{if } k = 1, \forall j \in (1, \ldots J) \\ z_j + \epsilon & \text{if } k = 2, j \notin \texttt{D} \\ znew_j + \epsilon & \text{if } k = 2, j \in \texttt{D} \end{cases}$$

where $\epsilon \sim N(0, 0.01)$ and

$$znew_j = \begin{cases} 0.20 * N(\mu_1, \sigma_1^2) + 0.80N(\mu_2, \sigma_2)^2 & \text{if } S_j = 0 \\ N(\mu_0, \sigma_0)^2 & \text{if } S_j = \{1, 2\} \end{cases}$$

Then, we can compute θ_{jk} as

$$\theta_{jk} = \frac{1}{1 + \exp(z_{jk})} \tag{10}$$

The expected number of methylated and unmethylated molecules at jth CpG site is $x_{jk}^M = \theta_{jk} N$ and $x_{jk}^U = (1-\theta_{jk})N$ where N is a scaling factor (e.g. $N = 10^6$).

3.2.1 Microarrays

To model saturation probe effect, we use the Langmuir adsorption model [16]. The "intensity" of methylated and unmethylated probes observed can be modeled using

$$M_{ijk} = o_{ijk} + d_{ijk} + a_{ijk}(\frac{x_{jk}^m}{x_{jk}^m + b_{ijk}})\epsilon_{ijk}$$

$$U_{ijk} = o_{ijk} + d_{ijk} + a_{ijk}(\frac{x_{jk}^u}{x_{jk}^u + b_{ijk}})\epsilon_{ijk}$$

where $i \in (1, \ldots, n_k)$ represents the sample, $j \in (1, \ldots, J)$ represents the CpG site (or probe) and $k \in (1, \ldots, K)$ represents the group index. Let T = total number of samples.
To calculate the "beta"-values which are values between 0 and 1 (where 1 is highly methylated), use

$$\beta_{ijk} = \frac{M_{ijk}}{M_{ijk} + U_{ijk} + \text{offset}} \tag{11}$$

where offset is a value to prevent dividing by 0 (e.g. offset = 100 which is the default from Illumina). Then we can compare the β_{ijk}'s to the true proportion of methylation θ_{jk} for the jth probe in the kth group.

We define the optical noise (o_{ijk}) to be written as a product of sample-level optical noise (o_{ik}) and probe-level optical noise (o_j) or $o_{ijk} = o_{ik} * o_j$ to allow for global shifts between samples. Both optical noise parameters are simulated using a lognormal normal distribution. Similarly, the florescence intensity from the scanner ($a_{ijk} = a_{ik} * a_j$) and the parameter ($b_{ijk} = b_{ik} * b_j$) are also a product of sample-level parameter and probe-level parameter to allow for global scaling between samples. The background noise (d_{ijk}), measurement error (ϵ_{ijk}) are simulated using a lognormal distribution. Table 3 contains a list of the example parameters used to simulate DNA methylation samples using microarrays. Each of the parameters sample-level noise (a_{ik}) and probe-level noise parameters (a_j) have their own hyperparameters to allow for global shifts. Similarly, $b_{ijk} = b_{ik} * b_j$ and $o_{ijk} = o_{ik} * o_j$ (optical noise) with their own set of hyperparameters.

Using the multivariate normal distribution, we simulate $\log_2(a)$, $\log_2(b)$ and $\log_2(o)$ with a given set of hyperparameters (see Table 3). In the `quantroSim` R-package, the variance hyperparameters are referred to as `siga`, `sigb` and `sigOpt` in the `simulateMeth()` function. In `quantroSim`, the level of technical variation induced from the platform technology is controlled using the covariance matricies of `siga`, `sigb` and `sigOpt` in the function `simulateMeth()`. There is a vignette available in the `quantroSim` R-package that contains more details about the `simulateMeth()` function. Here we define "low" technical variation as the default parameters in the functions

`simulateMeth()` (Table 3) and “high” technical variation as ten fold increase for `siga` and `sigb` and a two fold increase for `sigOpt`. An example of simulated DNA methylation arrays with “low” and “high” technical variation and default parameters otherwise is given in Figure 19 and 20, respectively.

```
library(quantroSim)
set.seed(999)
siga = sigb = 0.1 * diag(10)
sigOpt = 1 * diag(10)
methTruth <- simulateMethTruth(nProbes = 1e4, nGroups = 2,
                               pDiff = 0.05, pUp = 0.80, verbose = FALSE)
sim <- simulateMeth(methTruth, meth.platform = "methArrays", nSamps = 5,
                    siga = siga, sigb = sigb, sigOpt = sigOpt,
                    nMol = 10^6, verbose = FALSE)
plotMeth(sim)
```

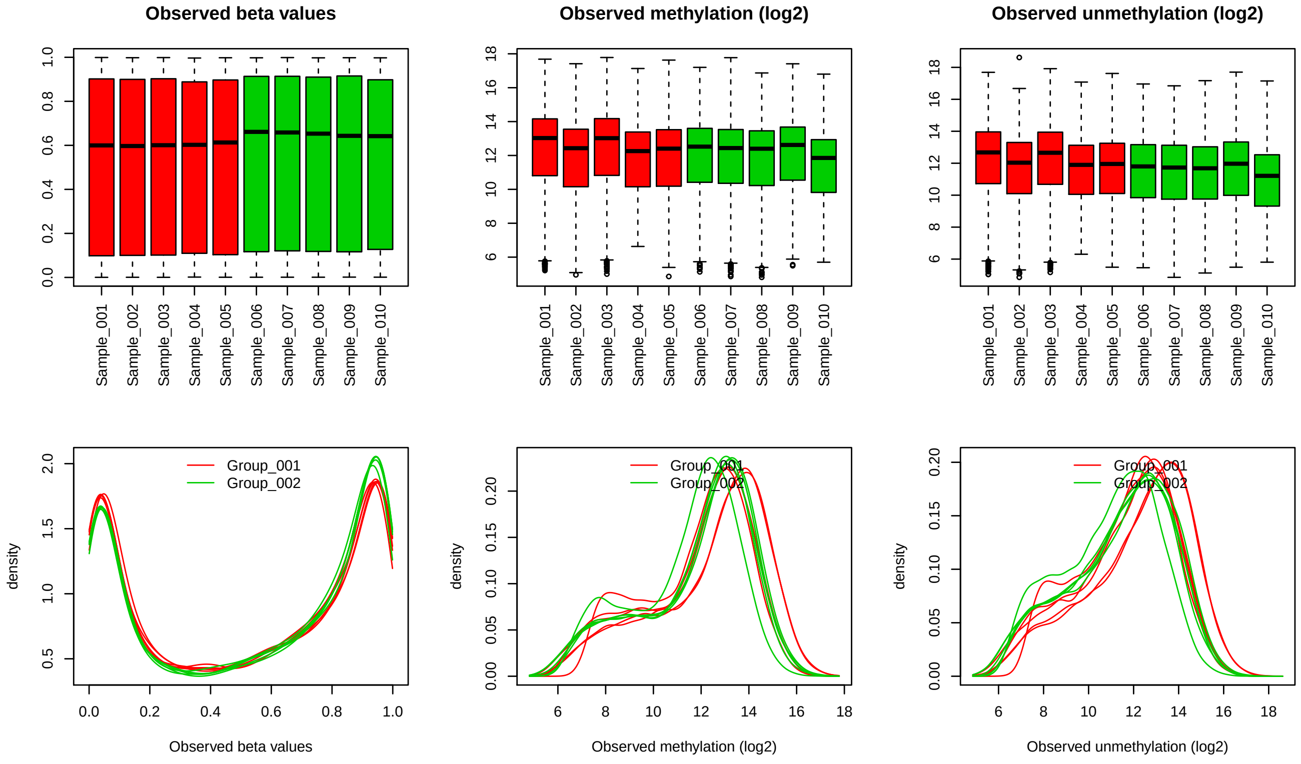

Figure 19: An example using the `simulateMethTruth()` and `simulateMeth()` functions in the `quantroSim` package to simulate 10 DNA methylation arrays each with 10000 CpGs where 5% of the CpGs are differentially methylated two groups (5 samples in each group). The default parameters in `simulateMeth()` are used for the level of technical variability.

```r
set.seed(999)

siga = sigb = 1 * diag(10)

sigOpt = 2 * diag(10)

methTruth <- simulateMethTruth(nProbes = 1e4, nGroups = 2,
                               pDiff = 0.05, pUp = 0.80, verbose = FALSE)

sim <- simulateMeth(methTruth, meth.platform = "methArrays",
                    nSamps = 5, nMol = 10^6, siga = siga,
                   sigb = sigb, sigOpt = sigOpt, verbose = FALSE)

plotMeth(sim)
```

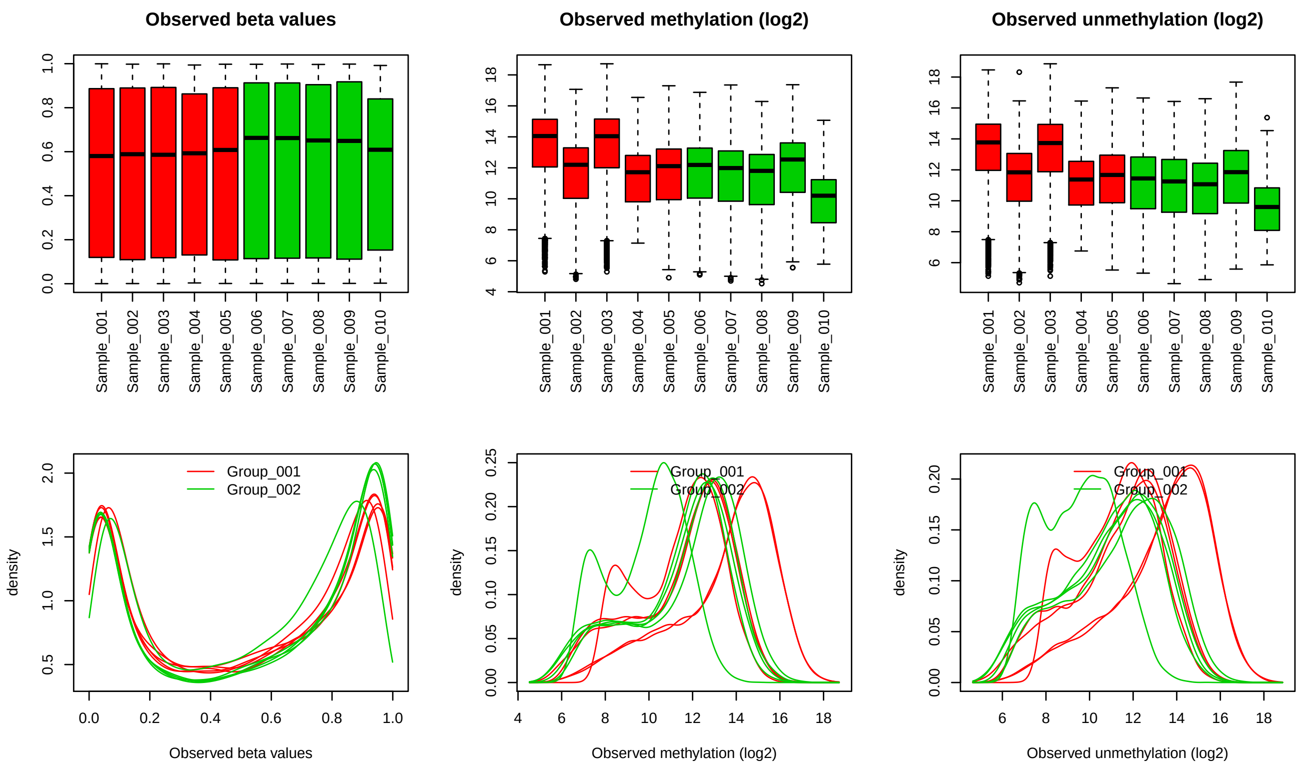

Figure 20: An example using the `simulateMethTruth()` and `simulateMeth()` functions in the `quantroSim` package to simulate 10 DNA methylation arrays each with 10000 CpGs where 5% of the CpGs are differentially methylated two groups (5 samples in each group). The parameters of `siga`, `sigb` and `sigOpt` were increased to increase the level of technical variability.

Table 3: Default parameters for distributions to simulate DNA methylation samples using microarrays.

	Parameter	Distribution
sample-level	$\log_2(a_{ik})$	$N(16, 0.1)$
	$\log_2(b_{ik})$	$N(22, 0.1)$
	$\log_2(o_{ik})$	$N(5, 1)$
probe-level	$\log_2(a_j)$	$N(0, 0.1)$
	$\log_2(b_j)$	$N(0, 0.1)$
	$\log_2(o_j)$	$N(0, 0.1)$
	$\log_2(d_j)$	$N(5, 1)$
	$\log_2(\epsilon_j)$	$N(0, 1)$

4 Simulation Study: Assessing the Performance of `quantro`

In the previous section, we discussed the `quantroSim` R-package that we developed to simulate gene expression and DNA methylation data. Here, we evaluate the performance of global normalization methods in the context of targeted and global changes in distributions with the goal of detecting differentially methylated CpGs. We performed a Monte Carlo simulation study to illustrate how the use of global normalization methods, such as quantile normalization, is not always appropriate. For the simulation study, we simulate DNA methylation arrays with a goal of detecting differentially methylated CpGs, but note these results also translate for differential gene expression. We compare naively using quantile normalization to using `quantro` to guide the decision of using either quantile normalization or no normalization to assess the cost of using global normalization methods in the context of distributions with global differences. We performed several simulation studies to evaluate the bias, mean squared error (MSE), false discovery rate (FDR), true positive rate (TPR) and false positive rate (FPR) (see Table 4 for a description of the performance metrics).

For the following simulation studies, we simulate DNA methylation arrays with a goal of detecting differentially methylated CpGs, but note these results also translate for differential gene expression. We consider two groups with five samples (total of 10 samples). For each set of 10 simulated samples, we control `pDiff` (the proportion of CpGs differentially methylated between the two groups). Once a set of 10 DNA methylation samples are simulated, we process the raw "beta"-values using both quantile normalization (using the function `normalize.quantiles()` in the `preprocessCore` [18] R/Bioconductor package) and `quantro`. The function `quantro()` in the `quantro` R/Bioconductor package uses the $F_{quantro}$ test statistic and the significance level α in a permutation test to assess the statistical significance:

$$\text{If } p = \sum_{b=1}^{B} I_{[F^b_{quantro} > F_{quantro}]} \geq \alpha \quad \Rightarrow \quad \text{Quantile normalization}$$

$$\text{If } p = \sum_{b=1}^{B} I_{[F^b_{quantro} > F_{quantro}]} < \alpha \quad \Rightarrow \quad \text{No normalization}$$

Finally, we estimate the difference in group means between the two groups and find the top differently methylated probes using a t-test (specifically `rowttests()` in the `genefilter` [19] R/Bioconductor package for efficiency). The plots containing the results from the simulation studies were created using the `ggplot2` R package [20].

4.1 Performance metrics

We considered five performance metrics to assess the performance of processing the raw data with quantile normalization compared to using `quantro` to decide if quantile normalization is appropriate (Table 4). In Section 3.2, we defined θ_{jk} (10) as the true "beta"-values at the jth probe and k^{th} group. We also defined the simulated "beta"-values β_{ijk} for the ith sample, j^{th} probe and k^{th} group (11). Here, we compute $\bar{\beta}_{\cdot j1}$ and $\bar{\beta}_{\cdot j2}$ which are the

"beta"-values averaged across the samples within each group at the jth probe to estimate the bias and MSE when detecting differentially methylated CpGs after using `quantro` or just quantile normalization.

The false discovery rate (FDR), true positive rate (TPR) and false positive rate (FPR) are computed using the notation of true positives (TP), false positives (FP), false negatives (FN) and true negatives (TN). We selected the top differentially methylated CpGs using p-values from a t-test that have been adjusted using the Benjamini and Hochberg adjustment to correct for multiple testing (`p.adjust()` function in the `stats` R-package). The number of top CpGs selected is the threshold that is varied to compute FDR, TPR and FPR. The false discovery rate (FDR) is calculated using as the number of incorrectly selected CpGs from a given set of top differentially methylated CpGs. The true positive rate (TPR) is calculated as the number of correctly selected CpGs from the set of true differentially methylated CpGs. The false positive rate (FPR) is calculated as the number of incorrectly selected CpGs from the set of CpGs that are not differentially methylated. The receiver operating characteristic (ROC) curve is used to depict the relative trade-offs between TPR and FPR. We used partial area under the curve (pAUC) [21] to compare the ROC curves.

Table 4: Performance Metrics

Performance Metrics	Formula
Bias	$E\Big[\Big\lvert E_j[\lvert\bar{\beta}_{.j1} - \bar{\beta}_{.j2}\rvert] - \lvert\theta_{j1} - \theta_{j2}\rvert\Big\rvert\Big]$
MSE	$E\Big[E_j\Big[\big(\lvert\bar{\beta}_{.j1} - \bar{\beta}_{.j2}\rvert - \lvert\theta_{j1} - \theta_{j2}\rvert\big)^2\Big]\Big]$
FDR	FP / (FP + TP)
TPR	TP / (TP + FN)
FPR	FP / (FP + TN)

4.2 Bias-Variance trade-off

In this first simulation study, we illustrate the bias-variance tradeoff of using normalization methods with and without global adjustments in the context of distributions with and without global differences. We assessed the relative bias (bias from `quantro` to the bias from quantile normalization) and relative mean squared error (MSE) while varying the threshold α from `quantro` (Section 4.2.1) and for a fixed cutoff threshold (Section 4.2.2).

We repeat the following procedure $N = 1000$ times:

1. Define the proportion of differentially methylated CpGs (`pDiff`) as 0 if no differences between groups or randomly sample `pDiff` from a uniform distribution with parameters a and b: $U(a, b)$. Using the `simulateMethTruth()` and `simulateMeth()` functions in the `quantroSim` R-package, simulate 10 DNA methylation samples each with 10,000 CpGs where `pDiff` defines the proportion of the CpGs that are differentially methylated two groups (5 samples in each group). We consider two levels of technical variability "low" and "high" which are discussed in Figures 19 and 20. Default parameters are used unless stated otherwise.

2. Normalize the 10 samples using both `quantro` and quantile normalization where `quantro` uses the $F_{quantro}$ test statistic (8) to decide if quantile normalization is appropriate with a significance level of α (no normalization otherwise).

3. Compute the bias and MSE when using `quantro` and quantile normalization.

We average the bias and MSE across the $N = 1000$ simulations.

4.2.1 Bias-Variance trade-off as a function of the cutoff used by `quantro`

To determine which threshold should be used when assessing the statistical significance of $F_{quantro}$, we compared the relative bias and relative MSE while varying the cutoff threshold used by `quantro`. We also considered different ranges for `pDiff`. If `pDiff` $= 0$, then there no differentially methylated CpGs between the two groups. As `pDiff` increases, the proportion of differentially methylated CpGs increases. Figure 21 gives examples of the relative bias and relative MSE under four ranges of `pDiff`:

1. `pDiff` $= 0$
2. `pDiff` $\sim U(0, 0.05)$
3. `pDiff` $\sim U(0, 0.08)$
4. `pDiff` $\sim U(0, 0.10)$

When there are no differences between the groups, quantile normalization reduces the bias and MSE in detecting true differences between groups of samples as it removes unwanted technical variation. As the number of differentially methylated CpGs increases, quantile normalization will remove both the unwanted technical and interesting biological variation resulting in higher bias and MSE when detecting differential methylation. In contrast, `quantro` reduces the bias and MSE compared to using quantile normalization because the method is able to detect when there are global differences.

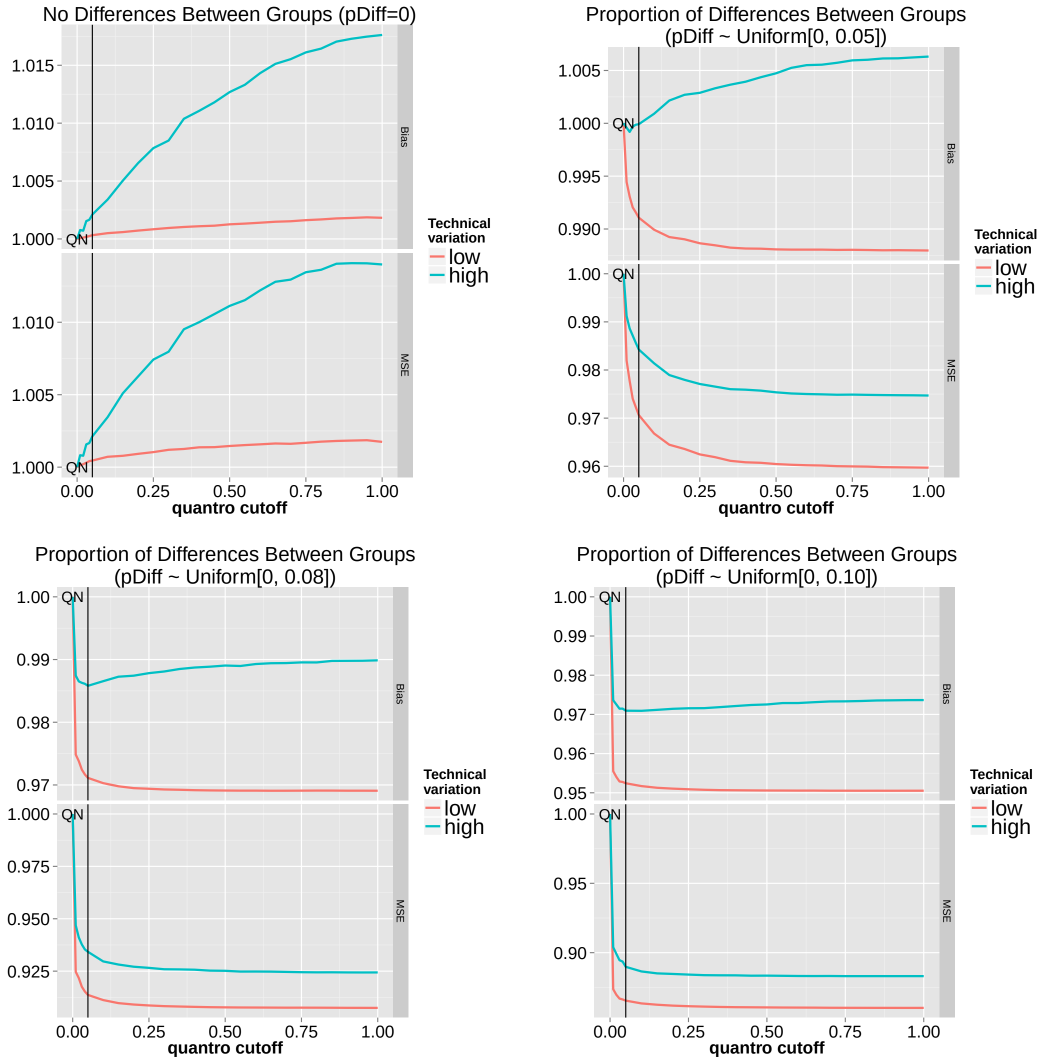

Figure 21: Bias-Variance trade-off as a function of the cutoff used by `quantro`. The figures show the relative bias (bias from `quantro` using a cutoff of $\alpha = 0.05$ to the bias from quantile normalization (QN)) and relative MSE. Differences between the distributions were simulated with four ranges: `pDiff` $= 0$ and `pDiff` $\sim U(0, 0.05)$, $U(0, 0.08)$, $U(0, 0.10)$ using low (pink) and high (blue) technical variation. The black vertical line is the $\alpha = 0.05$ cutoff.

4.2.2 Bias-Variance trade-off using `quantro` threshold of $\alpha = 0.05$

Here we focus on using one significance level from `quantro`. Using a significance level of $\alpha = 0.05$ (black line in Supplemental Figure 21), we compare the relative bias and relative MSE of `quantro` to quantile normalization. In Figure 22, we show that when there are global changes in distributions between the two groups, `quantro` reduces the bias and MSE compared to using quantile normalization because the method is able to detect when there are global differences.

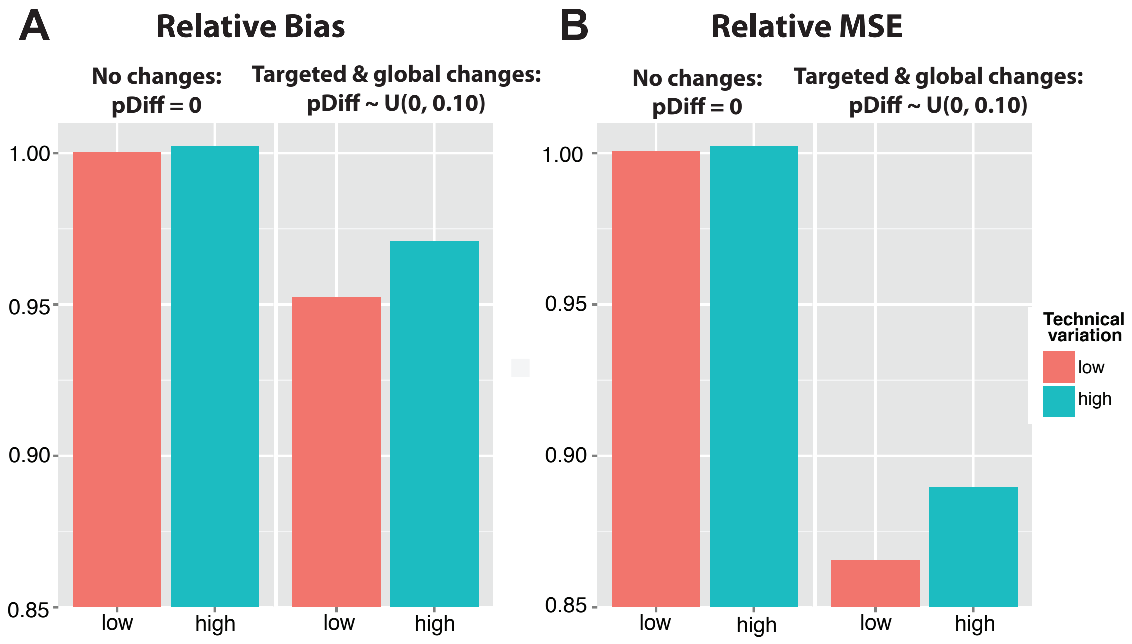

Figure 22: Bias-variance trade-off using quantro using cutoff of $\alpha = 0.05$. (A) Relative bias (bias from `quantro` using a cutoff of $\alpha = 0.05$ to the bias from quantile normalization) when considering no differentially methylated CpGs (`pDiff` $= 0$) and targeted and global changes in distributions between the two groups where `pDiff` was randomly sampled from a Uniform distribution ranging from 0 to 0.10. (B) Relative MSE (MSE from quantro using a cutoff of $\alpha = 0.05$ to the MSE from quantile normalization) with same `pDiff`.

4.3 Number of false discoveries

In the second simulation study, we estimate the number of false discoveries out of the top differentially methylated CpGs.

We repeat the following procedure $N = 100$ times:

1. Define the proportion of differentially methylated CpGs (`pDiff`) as one of the following: 0.01, 0.05, 0.10, 0.25. Using the `simulateMethTruth()` and `simulateMeth()` functions in the `quantroSim` R-package, simulate 10 DNA methylation samples each with 450,000 CpGs where `pDiff` defines the proportion of the CpGs that are differentially methylated two groups (5 samples in each group). Default parameters are used unless stated otherwise.

2. Normalize the 10 samples using both `quantro` and quantile normalization where `quantro` uses the $F_{quantro}$ test statistic (8) to decide if quantile normalization is appropriate with a significance level of α (no normalization otherwise).

3. Compute the number of false discoveries or false positives (FP) as a function of the number of top differentially

methylated CpGs selected (FP + TP) when using `quantro` and quantile normalization.

We average the number of false discoveries across the N simulations.

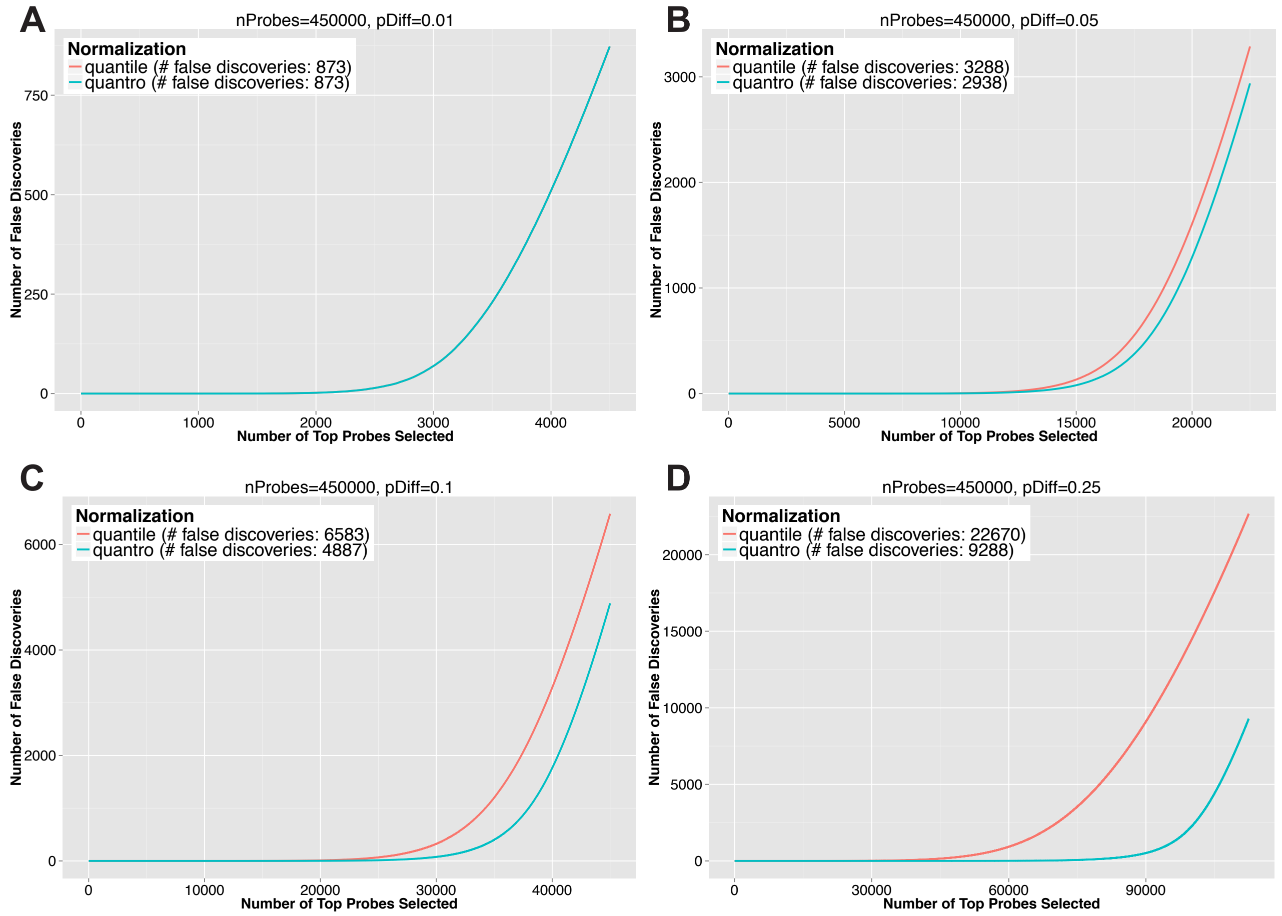

Figure 23: The number of false discoveries as a function of the number of top differentially methylated CpGs selected with a goal of detecting differentially methylated CpGs. Ten DNA methylation samples were simulated with 450K CpGs and an increasing proportion of differentially methylated CpGs between two groups: (A) `pDiff` = 0.01. (B) `pDiff` = 0.05. (C) `pDiff` = 0.10. (A) `pDiff` = 0.25.

4.4 Receiver operating characteristic curves

Here we illustrate the trade-off between the true positive rate (TPR) and the false positive rate (FPR) using an ROC curve. We measure the performance using partial area under the curve (pAUC).

We repeat the following procedure $N = 1000$ times:

1. Randomly sample the proportion of differentially methylated CpGs (`pDiff`) from a uniform distribution: Fig (A) $U(0, 0.05)$, Fig (B) $U(0, 0.10)$, Fig (C) $U(0, 0.25)$, Fig (D) $U(0, 0.50)$. Using the `simulateMethTruth()`

and `simulateMeth()` functions in the `quantroSim` R-package, simulate 10 DNA methylation samples each with 10,000 CpGs where `pDiff` defines the proportion of the CpGs that are differentially methylated two groups (5 samples in each group). Default parameters are used unless stated otherwise.

2. Normalize the 10 samples using both `quantro` and quantile normalization where `quantro` uses the $F_{quantro}$ test statistic (8) to decide if quantile normalization is appropriate with a significance level of α (no normalization otherwise).

3. Compute TPR and FPR as a function of the number of top differentially methylated CpGs selected (FP + TP) when using `quantro` and quantile normalization.

We average the TPRs across N simulations (similar for the FPRs). We compute the partial area under the curve (`pAUC`) as the area under the curve restricted to a false positive rate of less than 0.25.

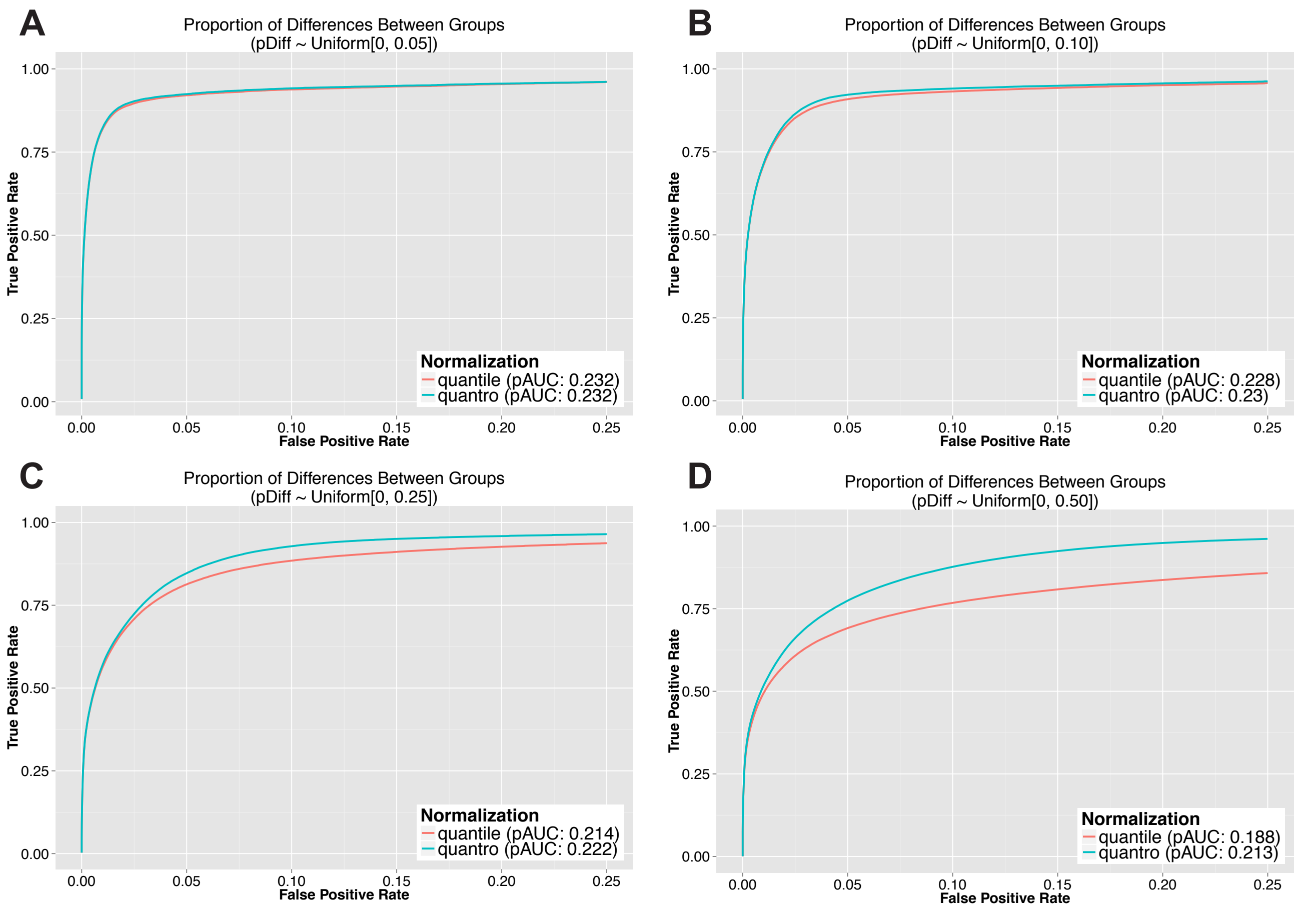

Figure 24: ROC curves depicting the trade-off between TPR and FPR with a goal of detecting differentially methylated CpGs as a function the number of top differentially methylated CpGs selected (FP + TP). The proportion of differentially methylated CpGs (`pDiff`) was randomly sampled from different Uniform distributions: (A) `pDiff` $\sim U(0, 0.05)$. (B) `pDiff` $\sim U(0, 0.10)$. (C) `pDiff` $\sim U(0, 0.25)$. (D) `pDiff` $\sim U(0, 0.50)$.

5 Alternatives to Global Normalization Methods

5.1 Application-specific normalization methods

If global adjustment methods are not appropriate, other methods such as *application-specific methods* [22] can be used. These are normalization methods where the adjustments are directly incorporated into the experiment or main analysis. Examples of these methods include the use of positive and negative control genes [23, 24, 22], the use of spike-in controls [25, 26, 27, 28, 29, 9, 30], and explicitly modeling known or unknown effects of unwanted variation in a linear model [31, 32, 33, 34, 35, 36]. Previous studies have evaluated and discussed normalization methods with and without global adjustments [37, 18, 38, 22, 39], but the decision of which type of normalization method to use depends on the outcome of interest.

5.1.1 Impact of experimental normalization in the context of global changes in gene expression

A recent study [9] discussed the use of normalization procedures in global gene expression analysis comparing two schematics: targeted changes in gene expression and global changes in gene expression such as transcriptional amplification [40, 41] or transcriptional shutdown [42]. The authors performed a gene expression experiment using P493-6 cells expressing low or high levels of c-Myc. Expression of high levels of c-Myc is considered to be a transcriptional amplification resulting in a 2-3 fold increase in RNA for each gene compared to cells with low levels of c-Myc. Therefore, the expression of high levels of c-Myc is expected to cause global changes in the distributions. As part of the experimental procedure, an experimental normalization was performed by introducing similar amounts of RNA from the two groups with global differences into the assay. The experiment consisted of four Affymetrix GeneChip arrays (GEO accession GSE40784) where 2 arrays were expressed low levels of c-Myc and 2 arrays expressed high levels of c-Myc. This data is the *mycAffyData* in Table 1.

We investigated if there were global differences in the distributions between the low and high c-Myc samples using the raw PM values from the original CEL files for this analysis. An AffyBatch object is available as R data package at `https://github.com/stephaniehicks/mycAffyData`. To visualize the true biological variation in the experimentally normalized samples, we divided the raw PM values by the sample mean of the PM values across the spike-ins on the log scale. Figure 25 plots the densities of the PM values before experimental normalization and the PM values from the CEL files (after experimental normalization). We tested for global differences in the distributions between the two groups using `quantro` using the PM values from the original CEL files (experimentally normalized samples). We assessed the statistical significance of the test statistic ($F_{quantro} = 1.548$) using permutation testing and report there were no global differences detected at the $\alpha = 0.05$ level between the distributions of the groups ($p = 0.318$).

Not surprisingly, the authors show global normalization methods are not appropriate if the total RNA is not the same across the samples. In this case, if normalization is performed at the experimental level (introducing

similar amounts of RNA into the assay from the two groups with global changes), then we suggest using control genes or spike-ins controls as no differences between the distributions will be detected (Figure 25). However, for the great majority of studies such strategies are not available. Furthermore, if one knows *a priori* that most genes are differentially expressed then it is not clear why one would use these high-throughput technologies.

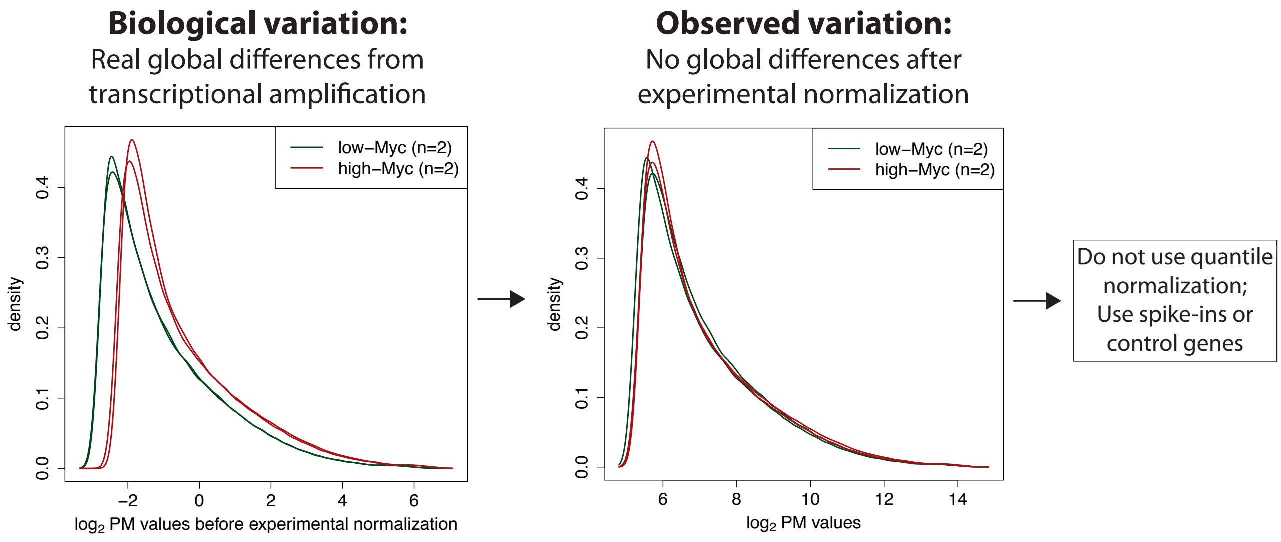

Figure 25: Global differences in distributions using $n = 4$ samples comparing expression of 'low' (green) and 'high' (red) levels of c-Myc which are expected to cause global biological changes in gene expression. No global differences in distributions are observed due to experimental normalization prior to measuring gene expression on microarrays. In this case, if normalization is performed at the experimental level by introducing similar amounts of RNA from the two groups with global differences into the assay, then we suggest to use control genes or spike-ins controls as no global differences between the distributions will be detected.

6 Software Available

The R-package quantro is available in Bioconductor 3.0 (http://www.bioconductor.org/packages/release/bioc/html/quantro.html) and the quantroSim R-package is available on Github (https://github.com/stephaniehicks/quantroSim).

References

[1] Mallows CL. "A note on asymptotic joint normality". In: *Annals of Mathematical Statistics* 43 (1972), pp. 508–515.

[2] Joseph K Pickrell et al. "Understanding mechanisms underlying human gene expression variation with RNA sequencing". In: *Nature* 464.7289 (2010), pp. 768–72. DOI: 10.1038/nature08872.

[3] Alyssa C Frazee, Ben Langmead, and Jeffrey T Leek. "ReCount: a multi-experiment resource of analysis-ready RNA-seq gene count datasets". In: *BMC Bioinformatics* 12 (2011), p. 449. DOI: 10.1186/1471-2105-12-449.

[4] Daniel Bottomly et al. "Evaluating gene expression in C57BL/6J and DBA/2J mouse striatum using RNA-Seq and microarrays". In: *PLoS One* 6.3 (2011), e17820. DOI: 10.1371/journal.pone.0017820.

[5] Prescott G Woodruff et al. "A distinctive alveolar macrophage activation state induced by cigarette smoking". In: *Am J Respir Crit Care Med* 172.11 (2005), pp. 1383–92. DOI: 10.1164/rccm.200505-686OC.

[6] Katrina Steiling et al. "A dynamic bronchial airway gene expression signature of chronic obstructive pulmonary disease and lung function impairment". In: *Am J Respir Crit Care Med* 187.9 (2013), pp. 933–42. DOI: 10.1164/rccm.201208-1449OC.

[7] Nicole M Lewandowski et al. "Polyamine pathway contributes to the pathogenesis of Parkinson disease". In: *Proc Natl Acad Sci U S A* 107.39 (2010), pp. 16970–5. DOI: 10.1073/pnas.1011751107.

[8] Markus Ahrens et al. "DNA methylation analysis in nonalcoholic fatty liver disease suggests distinct disease-specific and remodeling signatures after bariatric surgery". In: *Cell Metab* 18.2 (2013), pp. 296–302. DOI: 10.1016/j.cmet.2013.07.004.

[9] Jakob Lovén et al. "Revisiting global gene expression analysis". In: *Cell* 151.3 (2012), pp. 476–82. DOI: 10.1016/j.cell.2012.10.012.

[10] Tina Rönn et al. "A six months exercise intervention influences the genome-wide DNA methylation pattern in human adipose tissue". In: *PLoS Genet* 9.6 (2013), e1003572. DOI: 10.1371/journal.pgen.1003572.

[11] Tasnim Dayeh et al. "Genome-wide DNA methylation analysis of human pancreatic islets from type 2 diabetic and non-diabetic donors identifies candidate genes that influence insulin secretion". In: *PLoS Genet* 10.3 (2014), e1004160. DOI: 10.1371/journal.pgen.1004160.

[12] Lovisa E Reinius et al. “Differential DNA methylation in purified human blood cells: implications for cell lineage and studies on disease susceptibility”. In: *PLoS One* 7.7 (2012), e41361. DOI: 10.1371/journal.pone.0041361.

[13] Michael I Love, Wolfgang Huber, and Simon Anders. “Moderated estimation of fold change and dispersion for RNA-Seq data with DESeq2.” In: *bioRxiv doi:10.1101/002832* (2014).

[14] Laurent Gautier et al. “affy–analysis of Affymetrix GeneChip data at the probe level”. In: *Bioinformatics* 20.3 (2004), pp. 307–15. DOI: 10.1093/bioinformatics/btg405.

[15] Martin J Aryee et al. “Minfi: a flexible and comprehensive Bioconductor package for the analysis of Infinium DNA methylation microarrays”. In: *Bioinformatics* (2014). DOI: 10.1093/bioinformatics/btu049.

[16] Doeke Hekstra et al. “Absolute mRNA concentrations from sequence-specific calibration of oligonucleotide arrays”. In: *Nucleic Acids Res* 31.7 (2003), pp. 1962–8.

[17] Jean-Francois Mercier, Gary W Slater, and Pascal Mayer. “Solid phase DNA amplification: a simple Monte Carlo Lattice model”. In: *Biophys J* 85.4 (2003), pp. 2075–86. DOI: 10.1016/S0006-3495(03)74636-0.

[18] B M Bolstad et al. “A comparison of normalization methods for high density oligonucleotide array data based on variance and bias”. In: *Bioinformatics* 19.2 (2003), pp. 185–93.

[19] R Gentleman et al. *genefilter: methods for filtering genes from microarray experiments.* BioC 1.6 (R package version 1.46.1). 2005.

[20] Hadley Wickham. *ggplot2: elegant graphics for data analysis.* Springer New York, 2009.

[21] D K McClish. “Analyzing a portion of the ROC curve”. In: *Med Decis Making* 9.3 (1989), pp. 190–5.

[22] Johann A Gagnon-Bartsch and Terence P Speed. “Using control genes to correct for unwanted variation in microarray data”. In: *Biostatistics* 13.3 (2012), pp. 539–52. DOI: 10.1093/biostatistics/kxr034.

[23] J. Lucas et al. *Sparse statistical modeling in gene expression genomics.* New York, New York: Cambridge University Press, 2006.

[24] Zhijin Wu and Martin J Aryee. “Subset quantile normalization using negative control features”. In: *J Comput Biol* 17.10 (2010), pp. 1385–95.

[25] A A Hill et al. “Evaluation of normalization procedures for oligonucleotide array data based on spiked cRNA controls”. In: *Genome Biol* 2.12 (2001), RESEARCH0055.

[26] A. J. Hartemink et al. “Maximum likelihood estimation of optimal scaling factors for expression array normalization”. In: *P Soc Photo-Opt Ins* 2.132-140 (2001).

[27] Vladimír Benes and Martina Muckenthaler. “Standardization of protocols in cDNA microarray analysis”. In: *Trends Biochem Sci* 28.5 (2003), pp. 244–9. DOI: 10.1016/S0968-0004(03)00068-9.

[28] Ali Mortazavi et al. "Mapping and quantifying mammalian transcriptomes by RNA-Seq". In: *Nat Methods* 5.7 (2008), pp. 621–8. DOI: 10.1038/nmeth.1226.

[29] Lichun Jiang et al. "Synthetic spike-in standards for RNA-seq experiments". In: *Genome Res* 21.9 (2011), pp. 1543–51. DOI: 10.1101/gr.121095.111.

[30] Nicolas Bonhoure et al. "Quantifying ChIP-seq data: a spiking method providing an internal reference for sample-to-sample normalization". In: *Genome Res* 24.7 (2014), pp. 1157–68. DOI: 10.1101/gr.168260.113.

[31] W Evan Johnson, Cheng Li, and Ariel Rabinovic. "Adjusting batch effects in microarray expression data using empirical Bayes methods". In: *Biostatistics* 8.1 (2007), pp. 118–27. DOI: 10.1093/biostatistics/kxj037.

[32] Jeffrey T Leek and John D Storey. "Capturing heterogeneity in gene expression studies by surrogate variable analysis". In: *PLoS Genet* 3.9 (2007), pp. 1724–35. DOI: 10.1371/journal.pgen.0030161.

[33] Hyun Min Kang et al. "Variance component model to account for sample structure in genome-wide association studies". In: *Nat Genet* 42.4 (2010), pp. 348–54. DOI: 10.1038/ng.548.

[34] Jennifer Listgarten et al. "Correction for hidden confounders in the genetic analysis of gene expression". In: *Proc Natl Acad Sci U S A* 107.38 (2010), pp. 16465–70. DOI: 10.1073/pnas.1002425107.

[35] Brigham H Mecham, Peter S Nelson, and John D Storey. "Supervised normalization of microarrays". In: *Bioinformatics* 26.10 (2010), pp. 1308–15. DOI: 10.1093/bioinformatics/btq118.

[36] Andrew E Jaffe et al. "Bump hunting to identify differentially methylated regions in epigenetic epidemiology studies". In: *Int J Epidemiol* 41.1 (2012), pp. 200–9. DOI: 10.1093/ije/dyr238.

[37] John Quackenbush. "Microarray data normalization and transformation". In: *Nat Genet* 32 Suppl (2002), pp. 496–501. DOI: 10.1038/ng1032.

[38] André Fujita et al. "Evaluating different methods of microarray data normalization". In: *BMC Bioinformatics* 7 (2006), p. 469. DOI: 10.1186/1471-2105-7-469.

[39] Lana Xia Garmire and Shankar Subramaniam. "Evaluation of normalization methods in mammalian microRNA-Seq data". In: *RNA* 18.6 (2012), pp. 1279–88. DOI: 10.1261/rna.030916.111.

[40] Charles Y Lin et al. "Transcriptional amplification in tumor cells with elevated c-Myc". In: *Cell* 151.1 (2012), pp. 56–67. DOI: 10.1016/j.cell.2012.08.026.

[41] Zuqin Nie et al. "c-Myc is a universal amplifier of expressed genes in lymphocytes and embryonic stem cells". In: *Cell* 151.1 (2012), pp. 68–79. DOI: 10.1016/j.cell.2012.08.033.

[42] Ziv Bar-Joseph, Anthony Gitter, and Itamar Simon. "Studying and modelling dynamic biological processes using time-series gene expression data". In: *Nat Rev Genet* 13.8 (2012), pp. 552–64. DOI: 10.1038/nrg3244.
